# Targeting PNPO to suppress tumor growth via inhibiting autophagic flux and to reverse paclitaxel resistance in ovarian cancer

**DOI:** 10.1007/s10495-024-01956-3

**Published:** 2024-04-13

**Authors:** Xin Li, Wencai Guan, Huiqiang Liu, Jia Yuan, Fanchen Wang, Bin Guan, Junyu Chen, Qi Lu, Lingyun Zhang, Guoxiong Xu

**Affiliations:** 1 Research Center for Clinical Medicine, Jinshan Hospital, Fudan University, 1508 Longhang Road, Shanghai, 201508 China; 2 Department of Oncology, Shanghai Medical College, Fudan University, Shanghai, China; 3 Department of Obstetrics and Gynecology, Jinshan Hospital, Fudan University, Shanghai, China; 4 Department of Medical Oncology, Shanghai Geriatric Medical Center, Shanghai, China; 5 Department of Medical Oncology, Zhongshan Hospital, Fudan University, Shanghai, China; 6 Center for Tumor Diagnosis and Therapy, Jinshan Hospital, Fudan University, Shanghai, China

**Keywords:** Autophagy, Chemoresistance, Lysosome, Pyridox(am)ine-5'-phosphate oxidase, Reversal, Tumorigenesis

## Abstract

Our previous study showed that pyridoxine 5’-phosphate oxidase (PNPO) is a tissue biomarker of ovarian cancer (OC) and has a prognostic implication but detailed mechanisms remain unclear. The current study focused on PNPO-regulated lysosome/autophagy-mediated cellular processes and the potential role of PNPO in chemoresistance. We found that PNPO was overexpressed in OC cells and was a prognostic factor in OC patients. PNPO significantly promoted cell proliferation via the regulation of cyclin B1 and phosphorylated CDK1 and shortened the G2M phase in a cell cycle. Overexpressed PNPO enhanced the biogenesis and perinuclear distribution of lysosomes, promoting the degradation of autophagosomes and boosting the autophagic flux. Further, an autolysosome marker LAMP2 was upregulated in OC cells. Silencing LAMP2 suppressed cell growth and induced cell apoptosis. LAMP2-siRNA blocked PNPO action in OC cells, indicating that the function of PNPO on cellular processes was mediated by LAMP2. These data suggest the existence of the PNPO-LAMP2 axis. Moreover, silencing PNPO suppressed xenographic tumor formation. Chloroquine counteracted the promotion effect of PNPO on autophagic flux and inhibited OC cell survival, facilitating the inhibitory effect of PNPO-shRNA on tumor growth in vivo. Finally, PNPO was overexpressed in paclitaxel-resistant OC cells. PNPO-siRNA enhanced paclitaxel sensitivity in vitro and in vivo. In conclusion, PNPO has a regulatory effect on lysosomal biogenesis that in turn promotes autophagic flux, leading to OC cell proliferation, and tumor formation, and is a paclitaxel-resistant factor. These data imply a potential application by targeting PNPO to suppress tumor growth and reverse PTX resistance in OC.

**Supplementary Information:**

The online version contains supplementary material available at 10.1007/s10495-024-01956-3.

## Introduction

Ovarian cancer (OC) is the second most lethal gynecological malignancy worldwide [1]. In 2023, the estimated number of new cases and deaths of OC is 19,710 and 13,270, respectively, in the United States [2]. Epithelial ovarian cancer (EOC) represents over 90% of ovarian malignancies [3]. Because of the asymptomatic at the early stage, the majority of EOC patients are diagnosed at the advanced stage, leading to an unfavorable prognosis due to recurrence and chemoresistance [4]. The standard treatment for EOC is cytoreductive surgery combined with paclitaxel (PTX) and carboplatin. However, about 70% of patients with stage III-IV faced chemoresistance and relapsed within 3 years [3, 5]. Therefore, the development of reliable early detection methods and overcoming chemoresistance is crucial for improving the prognosis of patients with OC.

Pyridox(am)ine-5’-phosphate oxidase (PNPO) is an enzymic protein for the synthesis of pyridoxal 5’-phosphate (PLP), an active form of vitamin B6. Initially, most studies focus on the correlation of PNPO with epilepsy [6]. Later, our group found that PNPO is involved in tumorigenesis in ovarian and breast cancers [7, 8], which at least in part is regulated by the TGF-β signaling pathway [7, 9]. However, the mechanisms of tumor formation and chemoresistance remain unclear but have been inferred to be involved in various cellular processes, including autophagy, transport system, alteration of tubulin, cell cycle checkpoint, DNA damage system, oxidative stress, cancer stem cell, altered tumor microenvironment, etc. [10, 11]. It has been shown that autophagy, under physiological and pathological conditions, can sustain cell survival when facing stressful situations or in opposition and is related to tumor suppression by promoting cell apoptosis [12], in which lysosomes are involved in the degradation of unnecessary or improperly functioning components within a cell [13]. Concerning the action of autophagy in the progression of EOC, the development and discovery of autophagic inhibitors are taken into consideration for better treatment responses [14]. Chloroquine (CQ), one of the autophagic inhibitors, has been processed in clinical trials to evaluate its effect on the treatment of various diseases including cancers (https://www.clinicaltrials.gov/search?term=chloroquine). Initially, the combination of CQ and PTX has shown promising results. However, CQ shows side effects as well because it is a non-specific inhibitor of autophagy and can take unknown risks in vivo [15]. Therefore, more efforts need to be made to get a better understanding of the detailed role of autophagy in the progression of OC and its potential for a better therapeutic strategy.

Here we explored the function and mechanism of PNPO affecting tumor formation and PTX resistance via the process of lysosome-autophagic flux in OC.

## Methods and materials

### Cell culture

Human epithelial OC cell lines OVCAR-3, SK-OV-3, and A2780 were obtained from American Type Culture Collection (ATCC, Manassas, VA, USA) and were cultured in Roswell Park Memorial Institute-1640 (PRIM-1640), McCoy’5 A, and Dulbecco’s modified Eagle medium (DMEM, 4.5 g/L glucose) (Corning Inc., New York, USA), respectively, supplemented with 10% fetal bovine serum (FBS, Invitrogen, Carlsbad, CA, USA). Nontumorous human immortalized ovarian surface epithelial cell line IOSE-80, human embryonic kidney cell line HEK293T, and human PTX-resistant OC cell line A2780-PTX were obtained from FuHeng BioLogy (FuHeng BioLogy, Shanghai, China) and cultured in PRIM-1640 and DMEM, respectively, supplemented with 10% FBS. OVCAR-3-PTX (OV3R-PTX) was generated previously in our laboratory [16]. CQ (#C6628, Merk, Darmstadt, Germany) was used at a concentration of 50 nM. All cell lines were authenticated by short tandem repeat (STR) analysis and were routinely detected to be pathogen‑free and mycoplasma‑negative.

### Transfection of siRNA and plasmid and infection of shRNA

Small-interfering RNA (siRNA) of PNPO (si-PNPO) and negative control (NC) were synthesized by Shanghai GenePharma Co., Ltd (Shanghai, China). The sequences are listed in Table S1. Lentiviral-packaged short-hairpin RNA (sh-RNA) and mCherry-GFP-LC3 plasmid were purchased from BIOTECHNOLOGY (Shanghai, China). The PNPO-overexpressing plasmid was generated by cloning PNPO into the pcDNA3.1 vector (Invitrogen, Carlsbad, CA, USA). The X-treme GENE siRNA Transfection Reagent (Roche Applied Science, Indianapolis, USA) and Lipo8000 (Beyotime Biotechnology, Shanghai, China) were used for transfecting siRNA and plasmid, respectively.

### RNA isolation and quantitative real-time polymerase chain reaction (qRT-PCR)

Total RNA was extracted using an RNA-Quick Purification Kit (Yishan Biotechnology Co., Ltd, Shanghai, China). The complementary DNA (cDNA) was synthesized using a First Strand Complementary DNA Synthesis kit (Roche). cDNA was then amplified using the 7300 Real-Time PCR System (Applied Biosystems; Thermo Fisher Scientific, Inc., MA, USA) with the BeyoFast™ SYBR Green qPCR Mix (2X, High ROX (Beyotime). Actin was used as an internal control. Primer sequences are listed in Table S2.

### Protein extraction and Western blot

Total proteins were extracted using an SDS Lysis Buffer (Beyotime) supplemented with 1% phenylmethanesulfonylfluoride fluoride (Beyotime) and 1% phosphatase inhibitor (Nanjing KeyGen Biotech Co., Ltd, Nanjing, China). To separate nuclear and cytoplasmic proteins, the Minute™ SC-003 kit (Invent Biotechnologies, Inc., Beijing, China) was used according to the manufacturer’s instructions. Proteins were run on 10% SDS-PAGE gel and transferred into a PVDF membrane, followed by incubation with a primary antibody overnight. The following primary antibodies were used: rabbit anti-PNPO (1:2000 dilution), mouse anti-LAMP2 (1:2000 dilution), rabbit anti-TFEB (1:2000 dilution), and mouse anti-Actin (1:5000 dilution) from Proteintech (Proteintech Group, Inc, Wuhan, China); rabbit anti-cyclin B1 (1:2000 dilution) and rabbit anti-LC3A/B (1:1000 dilution) from Cell Signaling Technology, Inc. (Danvers, MA, USA); rabbit anti-Cathepsin G (CTSG, 1:2000 dilution) from Biorbyt Biotechnology, Inc. (Biorbyt Biotechnology, Inc., Wuhan, China); mouse anti-CDK1/CDK2 (1:2000 dilution) from Santa Cruz Biotechnology, Inc. (Santa Cruz Biotechnology, Inc., Dallas, USA); rabbit anti-CDK1 (phosphor T161) (1:2000 dilution) from Abcam (Cambridge, UK). Secondary antibodies were horseradish peroxidase-conjugated goat anti-rabbit IgG and anti-mouse IgG (both 1:10,000 dilution, Proteintech). Signals were detected using BeyoECL Moon (Beyotime) and quantified using ImageJ software (National Institutes of Health, USA).

### Immunofluorescence (IF)

For IF, cells were plated into glass dishes. After fixation with 4% paraformaldehyde (PFA) for 15 min, cells were permeabilized with 0.5% Triton X-100 in phosphate-buffered saline (PBS) for 15 min. After blocking with QuickBlock™ Immunostaining Block Solution (Beyotime) at room temperature for 1 h, cells were incubated with the mouse anti‐LAMP2 antibody (1:200 Dilution, Proteintech) at 4 °C overnight, followed by the secondary antibody Alexa Fluor 647‐conjugated goat anti-mouse IgG antibody (1:500 dilution, Cell Signaling Technology, Inc.) incubation at room temperature for 1 h. DAPI (Beyotime) was used for the stain of the nucleus. Cells were photographed under the BioTek Cytation C10 Confocal Image Reader (Agilent technologies, City, State) at ×200 or ×400 magnification.

### Cell viability, colony formation, EdU proliferation, and cell cycle assays

For the cell viability assay, pcDNA3.1-PNPO or si-PNPO transfected OVCAR-3 and SK-OV-3 cells were seeded in 96-well plates at the density of 4 × 10^3^ and 5 × 10^3^ per well, respectively, for 24, 48, and 72 h. Absorbance values for cells were measured using the CCK-8 kit (Beyotime) by BioTek Epoch (Winooski, VT, USA) at an optical density of 450 nm.

For colony formation, cells were plated into 6-well plates at a density of 1 × 10^3^/well. After 2 weeks, cells were fixed with 4% PFA for 30 min and stained with Crystal Violet Solution (Sigma-Aldrich Trading Co., Ltd, Shanghai) for 30 min. The number of colonies was analyzed using ImageJ software.

For the EdU (5-ethynyl-2’-deoxyuridine) proliferation assay, cells were seeded in a 24-well plate for 24 h and then labeled using the BeyoClick™ EdU-555 kit (Beyotime Biotechnology) according to the manufacturer’s instructions. Cells were photographed under the BioTek Cytation C10 Confocal Image Reader (Agilent Technologies) at ×200 magnification. The ratio of red/blue was analyzed using Image J software.

For cell cycle analysis, cells were seeded in a 6-well plate for 24 h. After trypsinization, cells were washed using cold PBS twice at 1,000 rpm for 5 min each and fixed with 70% ethanol at -20 ^0^C for 4 h. After washing cells with PBS twice and centrifugating, the cell pellet was resuspended in 500 µl propidium iodide (PI) solution (PI/RNase Staining Buffer, BD, New Jersey, USA) and incubated for 15 min in the dark. Fifteen thousand cells were acquired by flow cytometry (Beckman Coulter, Inc., Brea, CA, USA). Data were analyzed using Modfit software (Verity Software House, USA).

### Apoptosis assay

Cells were cultured in 6-well plates for 24 h. After harvesting, cells were washed with PBS and resuspended in 500 µl 1×binding buffer, 3 µl Annexin V (BD) and 5 µl PI were added to the cell suspension. After incubation for 15 min, the apoptotic cells were measured by flow cytometry (Beckman Coulter, Inc.).

### Paclitaxel cytotoxicity assay

Cells transfected with pcDNA3.1-PNPO plasmid or si-PNPO were seeded into a 96-well plate at a density of 5 × 10^3^ for 8 h, followed by adding PTX in gradient concentrations for 48 h. Finally, absorbance values for cells were measured using the CCK-8 kit (Beyotime) at 450 nm. The relative cell viability (%) was calculated as a percentage of living cell proportion for PTX-treated cells vs. untreated cells. The IC_50_ value was determined by the nonlinear regression analysis after the concentration of PTX was transformed to log(10).

### LysoTracker assay

Cells transfected with plasmid and siRNA were plated into a confocal glass bottom dish and incubated with LysoTracker (Beyotime Biotechnology) for 30 min at 37 °C. A confocal laser-scanning microscope (Leica SP5, Wetzlar, Germany) was used to capture the image.

### Three-dimensional (3D) culture

Cells were plated into an agarose gel-coated 96-well plate at the density of 400 cells/well. The sphereing images were captured every 2 days using a microscope (OLYMPUS, Tokyo, Japan). The diameter of the microsphere was analyzed and calculated as described previously [17].

### Spheroid formation experiment

Cells were plated into a 6-well ultra-low attachment culture plate (Corning Incorporated, Corning, NY, USA) at the density of 3000 cells/well, supplemented with 20 ng/ml epidermal growth factor (EGF, ThermoFisher, Waltham, MA, USA), 20 ng/ml basic fibroblast growth factor (bFGF, ThermoFisher), 4 µg/ml heparin (Sigma-Aldrich), and 0.4 µg/ml B27 (ThermoFishher) and chased every 3 days until day 12. The images were captured using a microscope (OLYMPUS, Tokyo, Japan). The diameter of the spheroid was analyzed and calculated as described previously [16].

### Xenograft mouse model

The study on animal subjects was approved by the Ethics Committee of Shanghai Public Health Clinical Center. Animals were sacrificed after anesthesia with 1% pentobarbital sodium intraperitoneal injection (80 mg/kg). For verifying the effect of CQ adjuvant therapy, a total of 8 × 10^6^ cells in 100 µl serum-free medium were subcutaneously injected into 5-week-old female BALB/c nude mice (*n* = 6/group, Shanghai Super-B&K Laboratory Animal Corp. Ltd., Shanghai, China). CQ (60 mg/kg) was administered by intraperitoneal injection every day for 2 weeks. For the PTX sensitivity assay, 6 × 10^6^ cells per mouse were injected subcutaneously into 5-week-old female nude mice (*n* = 8/group). PTX (15 mg/kg) was administered by intraperitoneal injection every 3 days for 3 weeks when the tumor reached a volume of approximately 80–100 mm^3^. Tumor volume was calculated as described previously [7].

### Immunohistochemistry (IHC) and hematoxylin and eosin (HE) staining

For IHC, tumor tissues were dissected from sacrificed mice. The tumor tissue was fixed in 10% neutral formalin. After paraffin embedding, the tissue specimen was sectioned (4 μm thick), deparaffinized in xylene for 5 min (thrice), and rehydrated in a descending alcohol series as in 100% anhydrous ethanol for 3 min (twice), in 90% and 70% ethanol for 3 min each. The sections were rinsed in water 3 times, placed in double-distilled water for 3 min, and then rinsed in PBS 3 times (3 min each). After blocking with 10% normal goat serum for 40 min at room temperature, the section was incubated with an anti-PNPO antibody (1:400 dilution, Proteintech), anti-Ki67 antibody (Proteintech), anti-LAMP2 antibody (1:600 dilution, Proteintech), or anti-LC3A/B antibody (1:1000 dilution, Cell Signaling) at 4 °C overnight, followed by incubation with Haopoly-HRP secondary antibody (Shanghai Jiehao Biotechnology, Inc, Shanghai, China) at room temperature for 1 h. Images were captured by OLYMPUS BX43 (OLYMPUS, Tokyo, Japan). Data were processed by the ImageJ IHC Profiler plugin automatically for the quantitative evaluation [18].

HE staining was used to evaluate the structure of the tumor tissue. The sections were stained in hematoxylin staining solution for 3–5 min and washed in double-distilled water. Next, sections were placed in 75% ethanol for 5 s, put into an eosin staining solution for 20 s, and washed once with double-distilled water. Then sections were placed sequentially in 75% and 95% ethanol for 1 min each and then dipped in xylene for 2 min to be transparent (twice). Finally, the sections were dried and sealed with neutral resin and observed under the microscope.

### TUNEL assay

The detection of apoptosis in tumor tissues from the xenograft mouse model was performed in deparaffined sections using the One-step TUNEL Apoptosis Detection Kit (Beyotime Biotechnology) according to the manufacturer’s instructions. TUNEL staining images were captured by a fluorescence microscope (OLYMPUS, Tokyo, Japan) and analyzed by ImageJ. The positive staining of the nuclei by DAPI (blue) and apoptotic cells (green) was presented in a percentage format.

### GEO data resource and clinical correlation analyses

Two Gene Expression Omnibus (GEO) expression profiling datasets (GSE17260 and GSE32062) containing normal ovarian samples and OC samples were downloaded from https://www.ncbi.nlm.nih.gov/geo/. Detailed information on the GEO series was summarized in Table S3. Overall survival (OS) of patients with the high/low expression of PNPO was analyzed based on the data from the above two GEO datasets using “survival”, “survminer”, “limma”, and “ggpubr” R-packages (https://github.com). The online UALCAN database (http://ualcan.path.uab.edu) was used to predict the correlation between PNPO expression and clinical features.

### Co-expression gene analysis and Gene Set Enrichment Analysis (GSEA) of PNPO

Co-expression genes of PNPO were extracted from TCGA-OV datasets (https://www.cancer.gov/ccg/research/genome-sequencing/tcga). The Database for Annotation, Visualization, and Integrated Discovery (DAVID 6.8) was used for the Gene Ontology (GO) enrichment analysis and Kyoto Encyclopedia of Genes and Genomes (KEGG) pathway enrichment analysis based on the above co-expression genes of PNPO. The enrichment results were then visualized using a “ggplot2” package (https://github.com) with a p-value < 0.05. GO enrichment analysis included biological processes (BP), cellular components (CC), and molecular function (MF) analyses. Samples from TCGA-OV were separated into high- and low-expression groups according to the median expression value of PNPO for the consequent GSEA and GSVA analysis. The GSEA of PNPO was performed based on the data from TCGA-OV. Gene Set Variation Analysis (GSVA) analysis was analyzed using the “GSVA” package (http://www.bioconductor.org) [19]. Gene Expression Profiling Interactive Analysis (GEPIA, http://gepia.cancer-pku.cn) was used to analyze the correlation between PNPO and LAMP2. A p-value < 0.05 and a false discovery rate (FDR) < 0.25 were considered as significant differences.

### PNPO expression-associated drug sensitivity analysis

NCI-60 compound activity data and RNA-seq expression profiles were acquired and downloaded from the CellMiner™ database (https://discover.nci.nih.gov/cellminer/home.do). The “impute”, “limma”, “ggplot2”, and “ggpubr” R packages (https://github.com) were used to analyze the correlation between PNPO expression and the half-maximal inhibitory concentration (IC_50_) of chemotherapeutic drugs in OC cells.

### Statistical analysis

All data were analyzed by IBM SPSS Statistics 27.0 (IBM SPSS Inc.), plotted by GraphPad Prism 8.0 (GraphPad Software Inc.), and presented as the mean ± SD. The student’s *t*-test was used for a two‐group comparison and one-way ANOVA followed by Turkey’s test was used for multiple comparisons with normal distribution and homogeneity of variance; otherwise, the Mann-Whitney test was used for a two-group comparison and the Kruskal-Wallis test was used for multiple comparisons. The Wilcoxon rank-sum test and Spearman rank test were applied to analyze the differences and correlations between the two groups, respectively. The Kaplan-Meier survival curve analysis was performed using the Log-rank test. The Cox hazard regression model was applied to calculate the hazard ratio (HR). Statistical significance was considered when *p* < 0.05.

## Results

### PNPO is overexpressed and is a prognostic factor in patients with OC

**Fig. 1:**
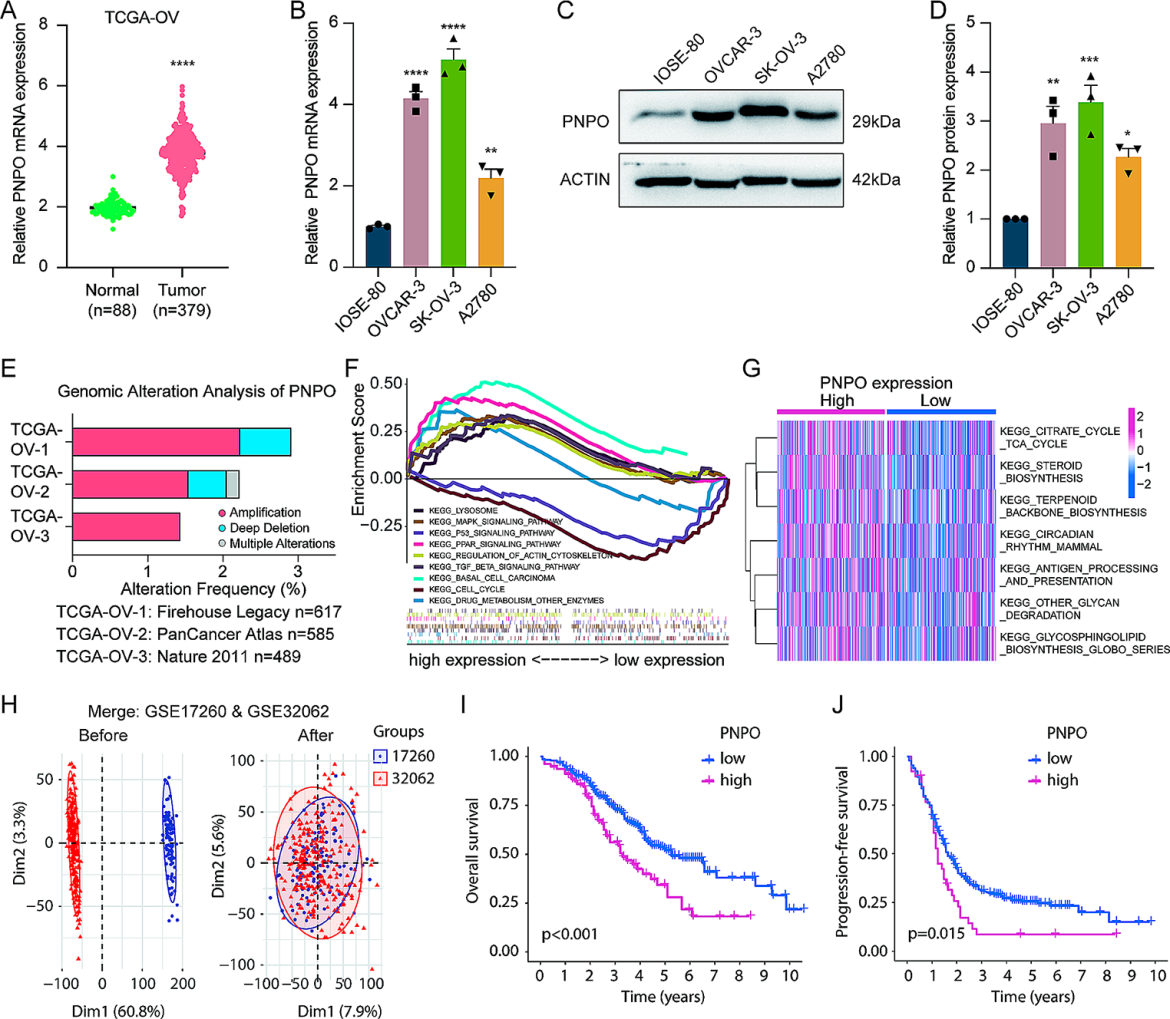
PNPO expression and biological function enrichment in ovarian cancer. **A** Comparison of PNPO mRNA expression between ovarian cancer and normal ovarian tissues. Data were extracted from the TCGA-OV database (https://www.cancer.gov/ccg/research/genome-sequencing/tcga) and the GTEx database (https://www.gtexportal.org/home/). The expression level of PNPO mRNA was higher in ovarian cancer than in normal ovary tissues. A P-value was calculated by the two-sided unpaired Student’s *t*-tes. **B** Expression of PNPO mRNA detected by qRT-PCR. Ovarian cancer cells (OVCAR-3, SK-OV-3, and A2780) versus non-cancerous immortalized ovarian surface epithelial cells (IOSE-80). Data were presented as mean ± SD (*n* = 3). A P-value was calculated by the one-way ANOVA followed by Tukey’s multiple comparisons test. **C** Expression of PNPO protein in OVCAR-3, SK-OV-3, A2780, and IOSE-80 cells detected by Western blot. Representative images are shown. **D** Semi-quantitative analysis of the protein bands in **C**. Data were presented as mean ± SD (*n* = 3). P value was calculated by the one-way ANOVA followed by Tukey’s multiple comparisons test. **E** Genomic alteration of PNPO in TCGA-OV database according to the cBioPortal website (https://www.cbioportal.org/). **F** GSEA analysis of OC samples with PNPO high- and low-expression from TCGA. Each line represents one particular gene set with a unique color. **G** GSVA analysis of PNPO expression correlated with variate gene sets. **H** Principal component analysis of GSE17260 and GSE32062 before and after removing the batch effect from individual datasets using the “limma” R package. **I** Association of PNPO with overall survival (OS) rate (*n* = 190 each at the beginning in PNPO high and low groups). **J** Association of PNPO with progression-free survival (*n* = 190 each at the beginning in PNPO high and low groups). *, *p* < 0.05; **, *p* < 0.01; ***, *p* < 0.001; ****, *p* < 0.0001

Compared with normal samples, PNPO mRNA was overexpressed in patients with OC based on the TCGA-OV database (Fig. 1A). qRT-PCR and Western blot confirmed that PNPO was higher in OC cells than in non-cancerous IOSE-80 cells at mRNA and protein levels (Fig. 1B-D). Genomic analysis showed that the amplification of PNPO was detected in OC patients (https://www.cbioportal.org/) (Fig. 1E). GSEA and GSVA analyses showed that the expression of PNPO in OC was correlated with some genes enriched in the lysosome, cell cycle, drug metabolism, TGF-β signaling pathway, and MAPK signaling pathway (Fig. 1F-G). Principal component analysis of GSE17260 and GSE32062 (https://www.ncbi.nlm.nih.gov/gds/) was processed before and after removing the batch effect from individual datasets for the subsequent Kaplan-Meier survival analysis (Fig. 1H) and the information on the GEO datasets was provided (Table S3). Kaplan-Meier Plotter analyses demonstrated that patients with a high level of PNPO had a low rate of OS and progression-free survival (Fig. 1I-J). Moreover, the biological function analyses of PNPO-associated genes were further replenished by GO (Fig. S1A-C) and KEGG (Fig. S1D). The GO term analyses showed the gene enrichments of the mitochondrial gene expression in biological process (BP), mitochondrial inner membrane, mitochondrial matrix, autophagosome in cellular component (CC), nucleocytoplasmic carrier activity in molecular function (MF), etc. KEGG signature also showed that PNPO-associated genes involved in the biosynthesis of cofactors, nucleocytoplasmic transport, etc.

### PNPO positively correlates with OC cell proliferation via the regulation of cyclin B1 and phosphorylated CDK1

**Fig. 2:**
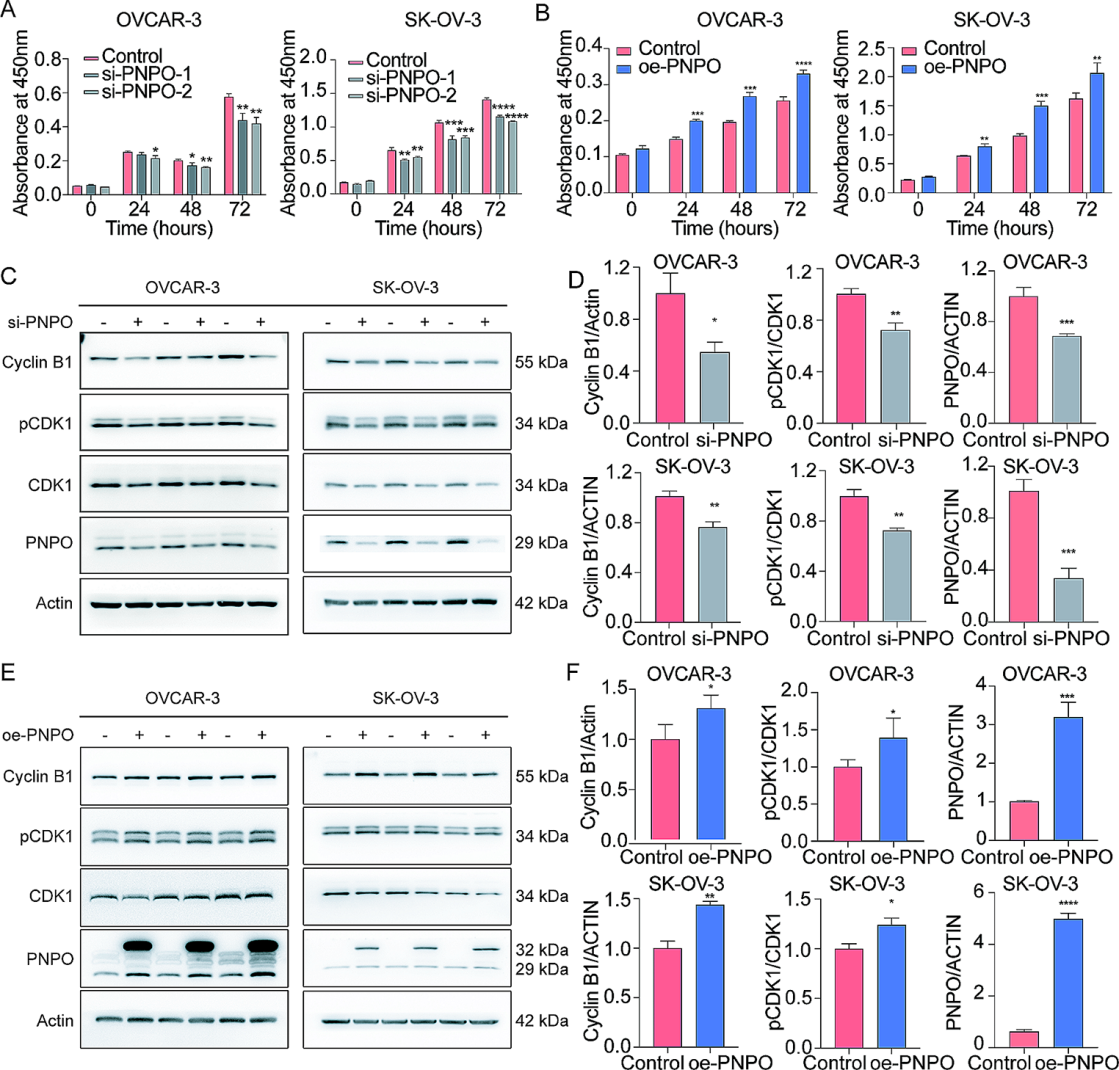
The effect of PNPO on OVCAR-3 and SK-OV-3 cell growth. **A** and **B** Detection of cell viability by CCK-8 assays. Negative control (Control), si-PNPO, or oe-PNPO transfected cells were seeded into a 96-well plate for 24, 48, and 72 h. **C** Detection of cyclin B1, phosphor-CDK1 (pCDK1), total CDK1, PNPO, and Actin proteins by Western blot after the knockdown of PNPO. Representative images are shown. **D** Semi-quantitative analysis of protein bands from C by the ratio of cyclin B1/Actin and pCDK1/CDK1. **E** Detection of cyclin B1, phosphor-CDK1 (pCDK1), total CDK1, PNPO, and Actin proteins by Western blot after the overexpression of PNPO. Representative images are shown. **F** Semi-quantitative analysis of protein bands from E by the ratio of cyclin B1/Actin and pCDK1/CDK1. Data were presented as mean ± SD (*n* = 3). P values were calculated by the one-way ANOVA followed by Tukey’s multiple comparisons tests (A) and by the two-sided unpaired Student’s *t*-test (B, D, and F). *, *p* < 0.05; **, *p* < 0.01; ***, *p* < 0.001; ****, *p* < 0.0001

Knockdown of PNPO decreased, whereas overexpression of PNPO increased, OVCAR-3 and SK-OV-3 cell viability detected by the CCK-8 assays (Fig. 2A-B). Validation of transfection efficacy of si-PNPO and oe-PNPO was proved by the detection of PNPO expression at mRNA and protein levels by qRT-PCR and Western blot, respectively (Fig. S2A-E). Subsequently, the cell cycle measurement by flow cytometry assay showed that overexpressing PNPO significantly shortened the G2M phase (Fig. S3A-B), indicating a more rapid transition of OC cells to mitosis, thus accelerating cell growth. Furthermore, Western blot confirmed that the knockdown of PNPO downregulated, whereas oe-PNPO upregulated, cell cycle-related proteins such as cyclin B1 and phosphorylated CDK1 (Fig. 2C-F). These data indicate that PNPO is positively correlated with OC cell proliferation.

### Overexpressed PNPO enhances the biogenesis and perinuclear distribution of lysosomes

**Fig. 3:**
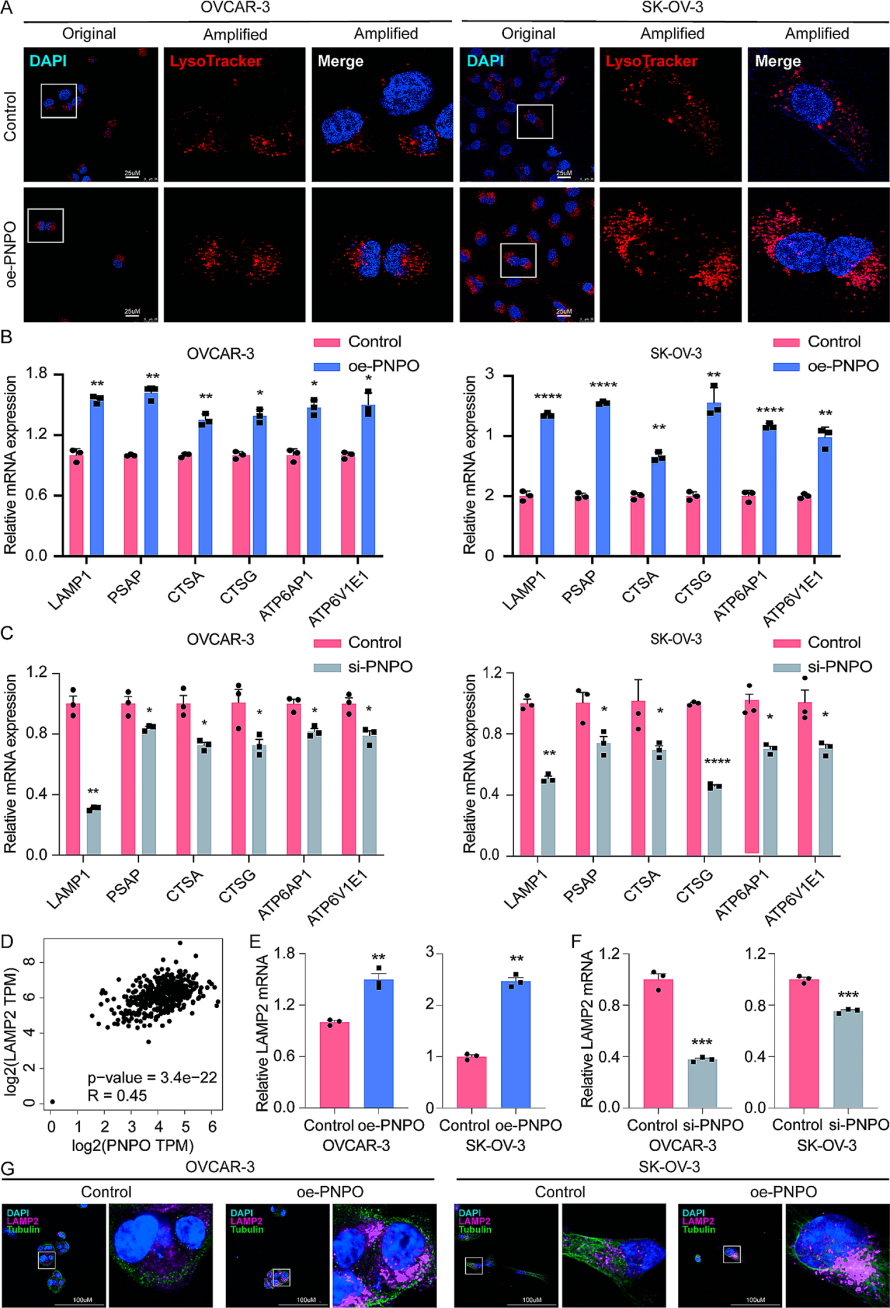
Effect of PNPO on the biogenesis and distribution of lysosomes in ovarian cancer cells. **A** Detection of lysosomes in OVCAR-3 and SK-OV-3 cells. Cells were plated into a 6-well plate and transiently transfected with PNPO-overexpressing plasmid for 48 h. The lysosomes were detected by the LysoTracker assay using red acidic probes. Scale bar, 25 μm. **B** Detection of lysosome-related mRNAs by qRT-PCR in cells after PNPO-overexpressing plasmid transfection. **C** Detection of lysosome-related mRNAs by qRT-PCR in cells after PNPO si-RNA transfection. **D** Correlation of PNPO with LAMP2 using the GEPIA2 (http://gepia.cancer-pku.cn). PNPO expression was positively correlated with LAMP2 expression. **E** Effect of PNPO on LAMP2 mRNA expression in ovarian cancer cells after PNPO-overexpressing plasmid transfection. **F** Effect of PNPO on LAMP2 mRNA expression in ovarian cancer cells after PNPO si-RNA transfection. **G** Detection of LAMP2 protein by immunofluorescence staining. PNPO-overexpressing cells were plated into a confocal dish for 48 h and LAMP2 was detected using the immunofluorescent assay. Scale bar, 100 μm. All assays were repeated at least three times. Data were presented as mean ± SD. P values were calculated by the two-sided unpaired Student’s *t*-test. oe-PNPO, PNPO-overexpressing plasmid; si-PNPO, PNPO si-RNA; *, *P* < 0.05; **, *P* < 0.01; ***, *P* < 0.001; ****, *p* < 0.0001

Since PNPO overexpression was detected to be associated with the lysosome pathway as shown in the GSEA (Fig. 1F), the LysoTracker assay was used to track the aggregation and distribution of lysosomes in OC cells. We found that oe-PNPO enhanced the red signals and increased perinuclear aggregation of lysosomes (Fig. 3A). Using gain-off-function and loss-of-function approaches, we found that increasing PNPO upregulated, whereas decreasing PNPO downregulated, lysosome PH-related, enzyme activity-related, membrane-related genes such as *LAMP1, PSAP, CTSA, CTSG, ATP6AP1*, and A*TP6V1E1*, respectively, in OVCAR-3 and SK-OV-3 cells (Fig. 3B-C). LAMP2, a canonical molecule presented on the membrane of the lysosome, was further analyzed using the GEPIA2 (http://gepia.cancer-pku.cn) and found to be positively correlated with PNPO (*p* < 0.0001) (Fig. 3D). Indeed, overexpression of PNPO increased, whereas knockdown of PNPO decreased, LAMP2 mRNA expression detected by qRT-PCR (Fig. 3E-F). Consequently, immunofluorescence staining confirmed LAMP2 protein expression in PNPO-overexpressing cells, suggesting that the overexpression of PNPO can induce the perinuclear aggregation of lysosomes (Fig. 3G). Western blot also showed that LAMP2 as well as CTSG, one of the lysosomal proteases partially representing the proteolytic function of lysosomes, was upregulated by oe-PNPO and downregulated by si-PNPO (Fig. S4A-B). Furthermore, Western blot analyses showed that the expression level of transcription factor EB (TFEB) in cell lysates was not affected by oe-PNPO transfection or si-PNPO transfection in SK-OV-3 cells (Fig. S5A-B). However, cytoplasmic and nuclear fraction analyses revealed that the level of TFEB was slightly decreased in the cytoplasm and increased in the nucleus by oe-PNPO and reversed by si-PNPO, while the phosphorylation of 14-3-3 protein, an active form of TFEB binding partner to inhibit TFEB nuclear translocation, was decreased by oe-PNPO and increased by si-PNPO in the cytoplasm of SK-OV-3 cells (Fig. S5C-D). These data demonstrated that PNPO promotes the translocation of TFEB from the cytoplasm to the nucleus by suppressing the phosphorylation of 14-3-3, thereby regulating LAMP2 expression.

### PNPO promotes the degradation of autophagosomes and enhances the autophagic flux in OC cells

**Fig. 4:**
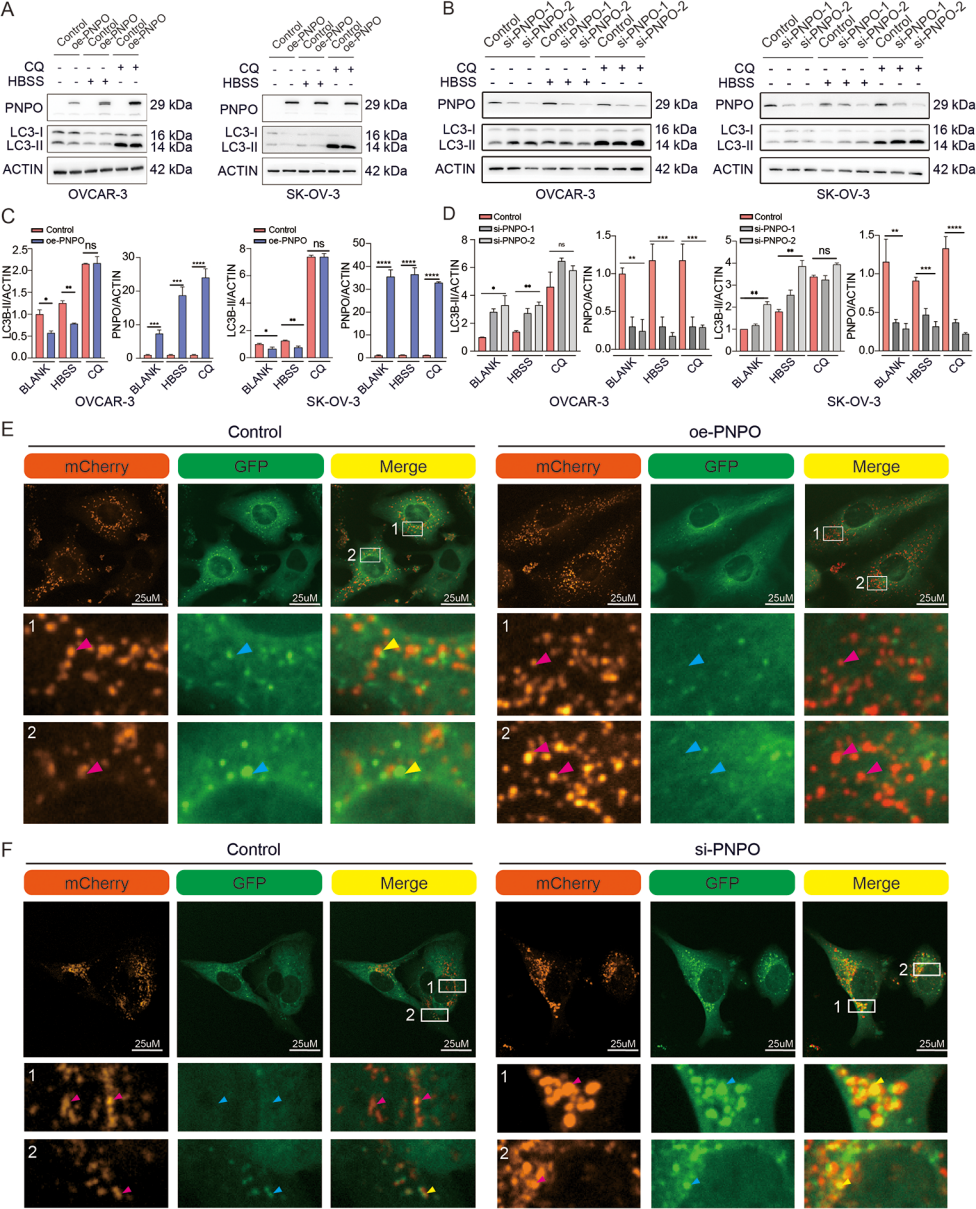
The effect of PNPO on autophagy in ovarian cancer cells. **A** and **B** Detection of autophagic protein LC3 in PNPO-overexpressing cells and PNPO-silencing cells. OVCAR-3 and SK-OV-3 cells were plated into 6-well plates after transfection of oe-PNPO plasmids (oe-PNPO) or PNPO-siRNA (si-PNPO) for 24 h. Cuture media were replaced with HBSS and incubated for 2 h or were supplemented with 50 µM CQ for 4 h before protein extraction. **C** Statistical analysis of (A) (*n* = 3). **D** Statistical analysis of (B) (*n* = 3). **E** and **F** Detection of autophagical flux in SK-OV-3 cells. Cells were transfected with mCherry-GFP-LC3 plasmid for 48 h and then replated into a confocal dish. The photos of cells were taken under a confocal microscope. The number in an image indicates an amplified zoom. Original amplification, x200. Scale bar, 25 μm. Assays were repeated at least three times. Data were presented as mean ± SD (*n* = 3). P values were calculated by the two-sided unpaired Student’s *t*-test (C). P values were calculated by the one-way ANOVA followed by Tukey’s multiple comparisons tests (D). ns, not significance versus the control group; *, *P* < 0.05; **, *P* < 0.01; ***, *P* < 0.001; ****, *p* < 0.0001

Overexpressed PNPO resulted in a decrease in autophagic protein LC3-II, whereas knockdown of PNPO resulted in an increase in LC3-II in OVCAR-3 and SK-OV-3 cells (Fig. 4A-D). The decreased LC3-II by oe-PNPO was also observed in cell culture by replacing the complete medium with Hank’s balanced salt solution (HBSS) for 2 h, which induced autophagy by the depletion of nutrients (Fig. 4A). Furthermore, the oe-PNPO-caused LC3-II decrease was abolished in the presence of a lysosomal inhibitor CQ in OC cells. The addition of CQ rescued the influence of PNPO on LC3-II, indicating that PNPO did not influence the formation of autophagosomes. These data suggest that PNPO regulates autophagy by influencing the degradation of autophagy rather than induction of autophagy. Interestingly, PNPO-promoted the perinuclear distribution and the hydrolytic function of lysosomes were observed. Using mCherry-GFP labeled LC3, we found that overexpressing PNPO led to a brighter red fluorescence (Fig. 4E), whereas silencing PNPO led to an increased yellow fluorescence (Fig. 4F), in SK-OV-3 cells. Because GFP is sensitive to acid which can degrade in acid conditions like lysosome, the GFP fluorescence may represent the dysfunction of lysosome in the process of degradation LC3. The perinucleus distribution of lysosomes is favorable for the degradation of autophagosomes. Thus, the red fluorescence alone represents a normal and enhanced autophagy flux after measurement, while the yellow fluorescence represents a decreased autophagy flux due to the impaired degradation process. These data indicate that PNPO enhanced the autophagy flux by increasing the function and perinucleus distribution of lysosomes.

### LAMP2 is upregulated in OC cells and is negatively associated with cell survival

**Fig. 5:**
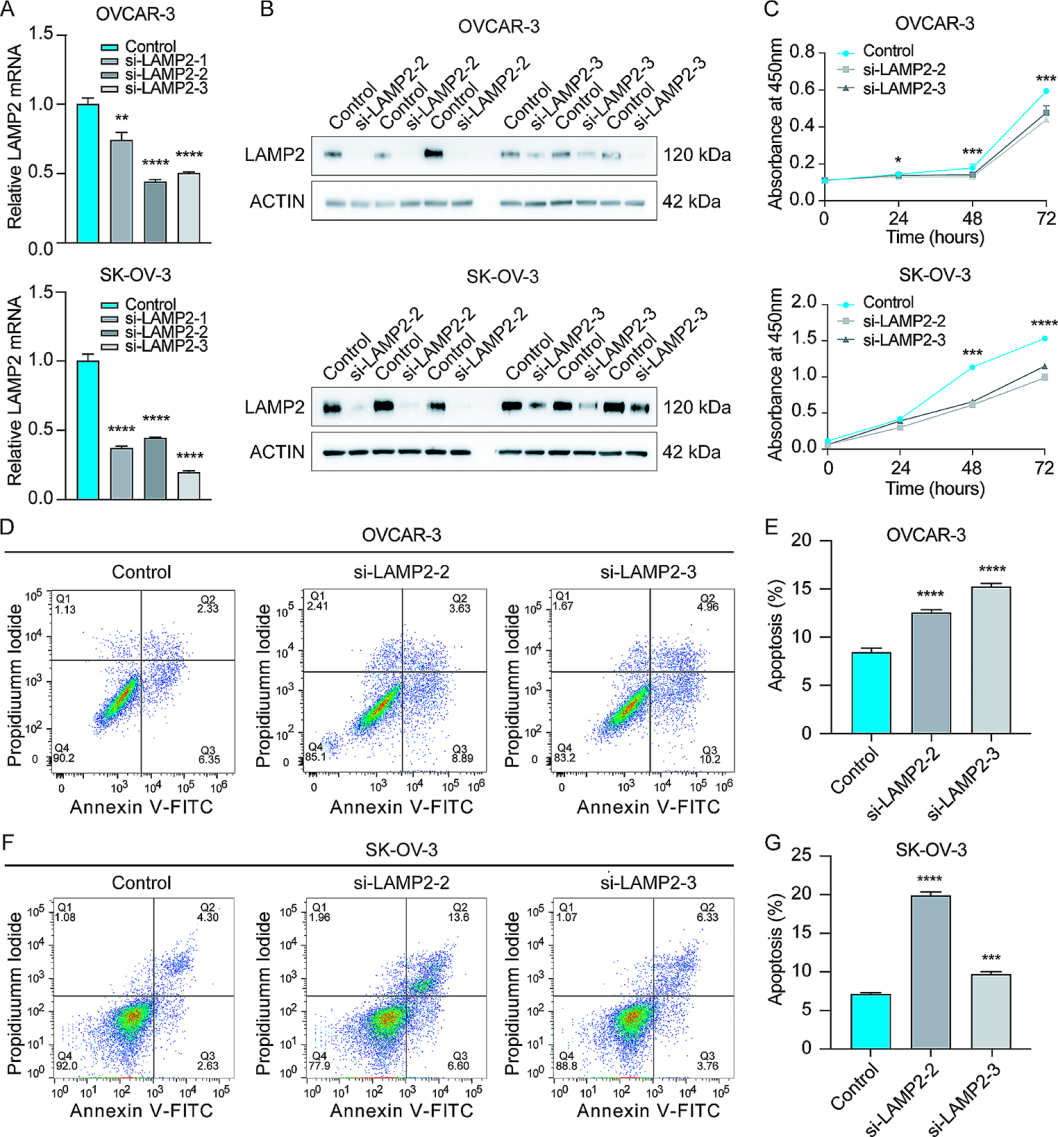
Effect of LAMP2-siRNA in OVCAR-3 and SK-OV-3 cells. **A** Detection of LAMP2 mRNA by qRT-PCR. Knockdown of LAMP2 was validated in cells transfected with 3 LAMP2-siRNAs for 48 h. **B** Detection of LAMP2 protein by Western blot in cells transfected with 2 LAMP2-siRNAs for 48 h. **C** Measurement of cell viability by the CCK8 assay. Cells were seeded into a 96-well plate and were transfected with negative control-siRNA or si-LAMP2 for 24 h. Silencing LAMP2 suppressed OC cell growth. **D-G** Detection of apoptosis. Cells were transfected with LAMP2-siRNA for 48 h and apoptotic cells were measured using flow cytometry. Data were presented as mean ± SD (*n* = 3). P values were calculated by the one-way ANOVA followed by Tukey’s multiple comparisons test. *, *P* < 0.05; **, *P* < 0.01; ***, *P* < 0.001; ****, *P* < 0.0001

Next, we examined lysosome protein LAMP2 expression and function in OC cells. The expression level of LAMP2 protein was higher in OC cell lines (OVCAR-3, and SK-OV-3) than in the non-tumorous ovarian surface cell line (IOSE-80) (Fig. S6A). Kaplan-Meier plot analyses of GSE26712, GSE8841, and GSE17260 datasets showed that high expression of LAMP2 was associated with poor prognosis of patients with OC (Fig. S6B-D). The efficiency of LAMP2 knockdown by 3 siRNAs was verified at RNA levels by qRT-PCR (Fig. 5A) and 2 siRNAs were confirmed at protein levels by Western blot (Fig. 5B). Consequently, the decrease in cell viability was observed in OC cells after LAMP2 knockdown (Fig. 5C). Flow cytometry analysis showed that apoptotic cells were increased after LMAP2 knockdown in OVCAR-3 and SK-OV-3 cells (Fig. 5D-H). These data demonstrated that silencing LAMP2 may suppress OC cell growth and induce OC cell apoptosis.

### Silencing LAMP2 blocks PNPO action in OC cells

**Fig. 6:**
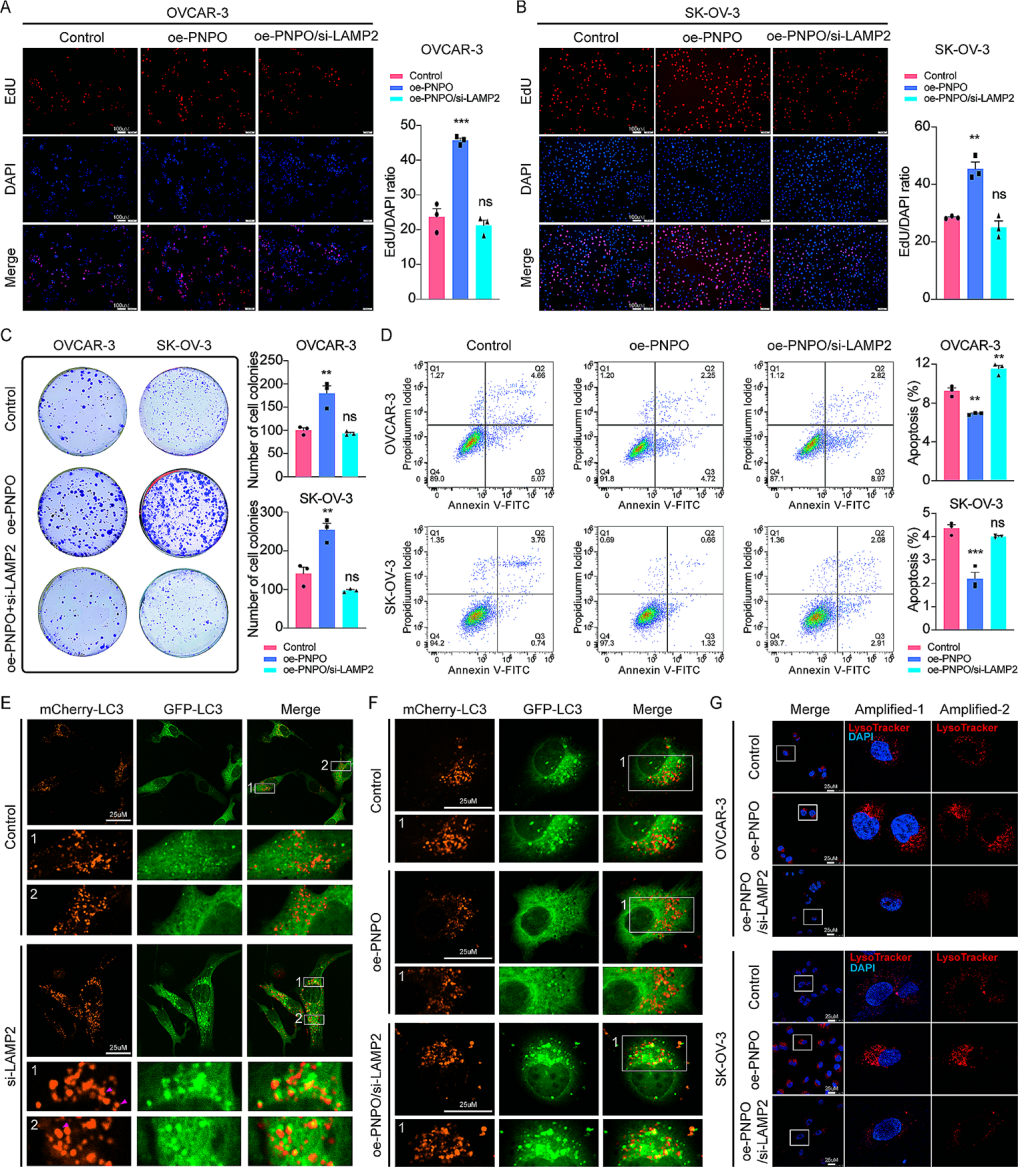
Effect of PNPO on ovarian cancer cell behaviors and the degradation of autophagy by LAMP2. **A** and **B** Detection of cell proliferation by the EdU assay. oe-PNPO-transfected OVCAR-3 and SK-OV-3 cells were seeded in a 24-well plate for 48 h in the presence or absence of si-LAMP2. The signal ratio of the EdU over the DAPI was calculated. Scale bar, 100 μm. **C** Measurement of colony formation. oe-PNPO-transfected OVCAR-3 and SK-OV-3 cells were seeded in a 6-well plate for 2 weeks in the presence or absence of si-LAMP2. **D** Detection of apoptosis by flow cytometry. oe-PNPO-transfected OVCAR-3 and SK-OV-3 cells were seeded in a 6-well plate for 48 h in the presence or absence of si-LAMP2. **E** Effect of si-LAMP2 on autophagy in SK-OV-3 cells. mCherry-GFP-LC3-infected cells were plated into a confocal dish for 48 h and the fluorescent signals were observed under a confocal microscope. Knockdown of LAMP2 hampered the degradation of autophagy. **F** Detection of si-LAMP2 effect on PNPO-induced autophagy in SK-OV-3 cells. The oe-PNPO and si-LAMP2 were co-transfected into mCherry-GFP-LC3-infected cells for 48 h. The promotion effect of PNPO on autophagy was impaired by the down-regulation of LAMP2. The number in an image indicates an amplified zoom. Original amplification, x200. **G** Detection of lysosomes. OVCAR-3 and SK-OV-3 cells were plated into 6-well plates, respectively, and transfected with oe-PNPO or co-transfected with oe-PNPO and si-LAMP2 for 48 h. Cells were then stained with LysoTracker for 30 min, followed by fluorescent microscopy. Original amplification, x400. Scale bar, 25 μm. The square area in an image was amplified in images amplified-1 and -2. PNPO promoted the perinuclear aggregation of lysosome which was counteracted by the silence of LMAP2. Assays were repeated at least three times. Data were presented as mean ± SD (*n* = 3). P values were calculated by the one-way ANOVA followed by Tukey’s multiple comparisons test. ns, not significance versus the control group; *, *P* < 0.05; **, *P* < 0.01; ***, *P* < 0.001

Next, we examined whether si-LAMP2 influences PNPO action on OC cell proliferation, colony formation, apoptosis, and autophagy. Overexpressing PNPO increased OC cell proliferation detected by the EdU assay and the oe-PNPO-induced proliferation was inhibited by the knockdown of LAMP2 in OVCAR-3 and SK-OV-3 cells (Fig. 6A-B). Colony formation assay further confirmed the effect of LAMP2 on PNPO action in these cells (Fig. 6C). The silencing of PNPO induced OVCAR-3 and SK-OV-3 cell apoptosis, whereas the overexpression of PNPO suppressed apoptosis (Fig. S7A-B), which was abolished in the presence of LAMP2-siRNA (Fig. 6D). These data suggest that PNPO-affected OC progression may be through autophagy. Furthermore, the knockdown of LAMP2 led to the dysfunction of lysosomes (Fig. 6E). Data from mCherry-GFP-LC3 plasmid transfection revealed that the increased autophagic flux by oe-PNPO was reversed in the presence of LAMP2-siRNA in SK-OV-3 cells (Fig. 6F). Similarly, the oe-PNPO-induced aggregation of lysosomes near the nucleus was blocked by the knockdown of LAMP2 (Fig. 6G). Generally, the above rescue assays indicate that the function of PNPO on cellular processes was potentially mediated by regulating autolysosome, precisely via LAMP2, proving the existence of the PNPO-LAMP2 axis.

### Knockdown of PNPO suppresses xenographic tumor formation by inhibiting autophagic flux

**Fig. 7:**
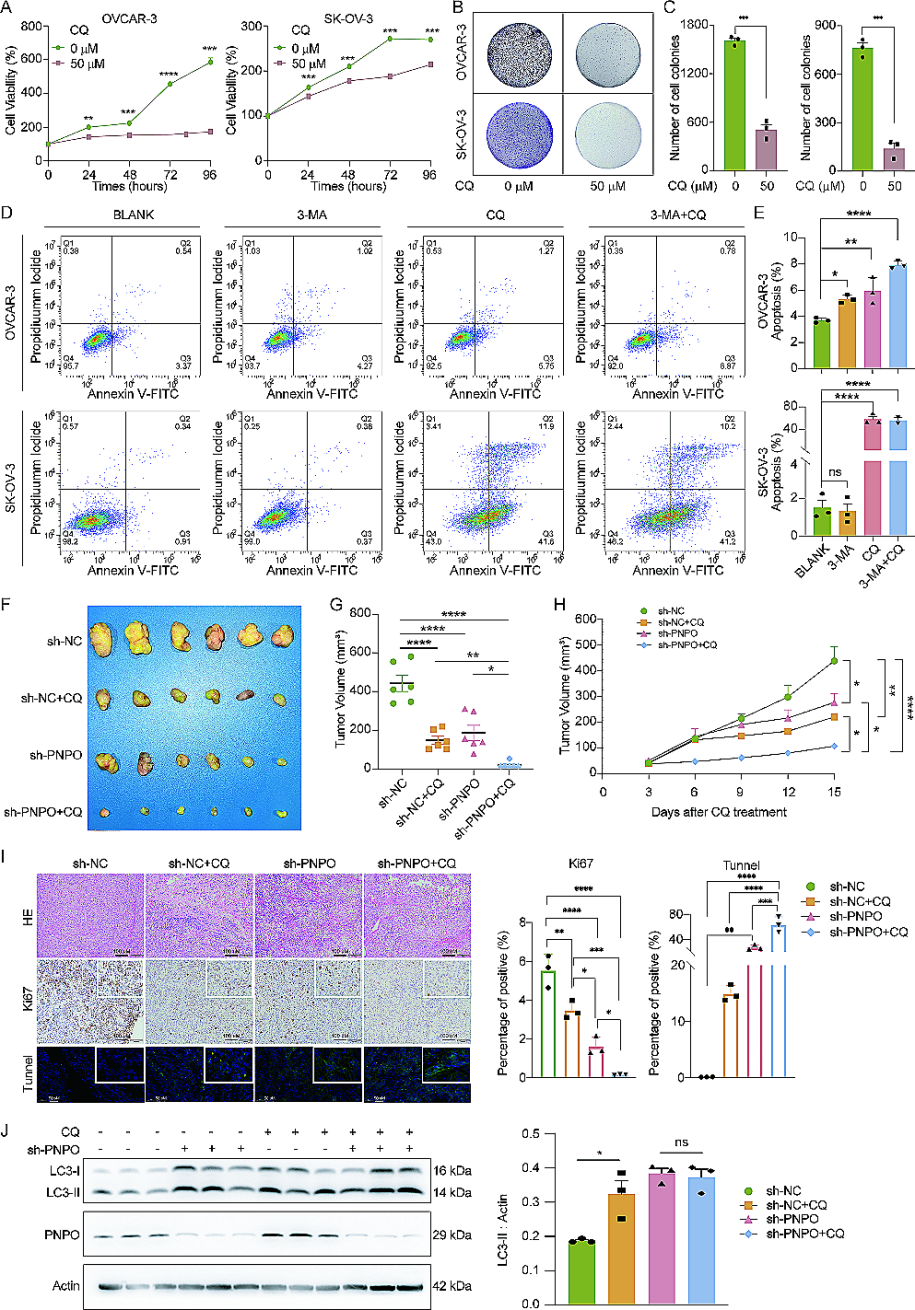
Facilitated effect of chloroquine (CQ) on the action of PNPO knockdown. **A** Effect of CQ on cell viability. OVCAR-3 and SK-OV-3 cells were treated with 50 µM CQ for 24, 48, 72, and 96 h and cell viability was measured by the CCK-8 assay. Data were presented as mean ± SD (*n* = 3). **B** Colony formation. OVCAR-3 and SK-OV-3 cells were plated in a 6-well plate and cultured in the presence or absence of 50 µM CQ for 14 days. **C** Quantitative analysis of B. The assay was repeated twice. Data were presented as mean ± SD from 3 wells. **D** Effect of CQ and 3-MA on cell apoptosis. OVCAR-3 and SK-OV-3 cells were treated with CQ (50 µM), 3-MA (5 mM), or both for 12 h and apoptotic cells were detected by flow cytometry. **E** Quantitative analysis of D. Data were presented as mean ± SD (*n* = 3). **F** Images of tumors. Nude mice were transplanted with sh-PNPO-infected SK-OV-3 cells. Each mouse was treated with 60 mg/kg of CQ and the tumor was removed at day 15 post-CQ administration and photographed. **G** Statistical analysis of the volume of tumors in F (*n* = 6). **H** Statistical analysis of the growth curve of tumors. **I** Detection of proliferating and apoptotic cells in tumor tissues. HE staining showed the structure of tumor tissue. IHC showed the expression of Ki67. Tunnel assay showed apoptotic cells. Scale bar, 100 μm. The lower panel is the statistical plot of Ki67 and Tunnel staining. Data were processed by the ImageJ IHC profiler plugin automatically and were presented as percentages of positive staining. **J** Detection of the effect of CQ on the expression of LC3 and PNPO in tumor tissues by Western blot. Data were presented as mean ± SD (*n* = 3). P values were calculated by two-sided unpaired Student’s *t*-test (A). P values were calculated by One-way ANOVA followed by Tukey’s multiple comparisons test (C, E, G, H, I, and J). ns, not significant; *, *P* < 0.05; **, *P* < 0.01; ***, *P* < 0.001; ****, *P* < 0.0001

Since PNPO promoted the degradation of autophagy, we subsequently examined the effect of PNPO on the relationship between tumor growth and autophagy in vitro and in vivo. Using CQ as an inhibitor of the autophagic flux, first of all, we found that 50 µM of CQ decreased OVCAR-3 and SK-OV-3 cell viability (Fig. 7A). Next, we also observed the inhibition of colony formation in OVCAR-3 and SK-OV-3 cells by CQ (Fig. 7B-C). Further, CQ dramatically increased OVCAR-3 and SK-OV-3 cell apoptosis, whereas 3-methyladenine (3-MA), a widely used inhibitor of autophagy by an inhibitory effect on phosphatidylinositol 3-kinases (PI3K), had weak effect on the apoptotic rate in OVCAR-3 cells and no effect in SK-OV-3 cells (Fig. 7D-E). An in vivo experiment showed that both the knockdown of PNPO and the administration of CQ dramatically inhibited SK-OV-3 cell xenotransplanted tumor growth, while the combination of sh-PNPO and CQ synergized the inhibitory effect (Fig. 7F). The statistical analysis of the tumor volume confirmed the significance of the inhibitory effect on tumor growth (Fig. 7G-H). HE staining showed the structure of tumor tissues and Ki67 staining and Tunnel assay demonstrated that the knockdown of PNPO decreased proliferating cells and increased apoptotic cells in tumor tissues (Fig. 7I), Additionally, Western blot revealed that the knockdown of PNPO and the administration of CQ stimulated an accumulation of LC3 by decreasing the degradation of LC3 in tumor tissues (Fig. 7J). Subsequent IHC showed that the expression of LC3 increased, while the expression of LAMP2 was decreased, in the PNPO knockdown group and further in the sh-PNPO/CQ-combined group (Fig. S8A-B). These data prove that the downregulation of PNPO can block the growth of a tumor and the administration of CQ can enlarge the inhibitory effect of sh-PNPO on tumor growth.

### PNPO is a paclitaxel-resistant factor in OC cells

**Fig. 8:**
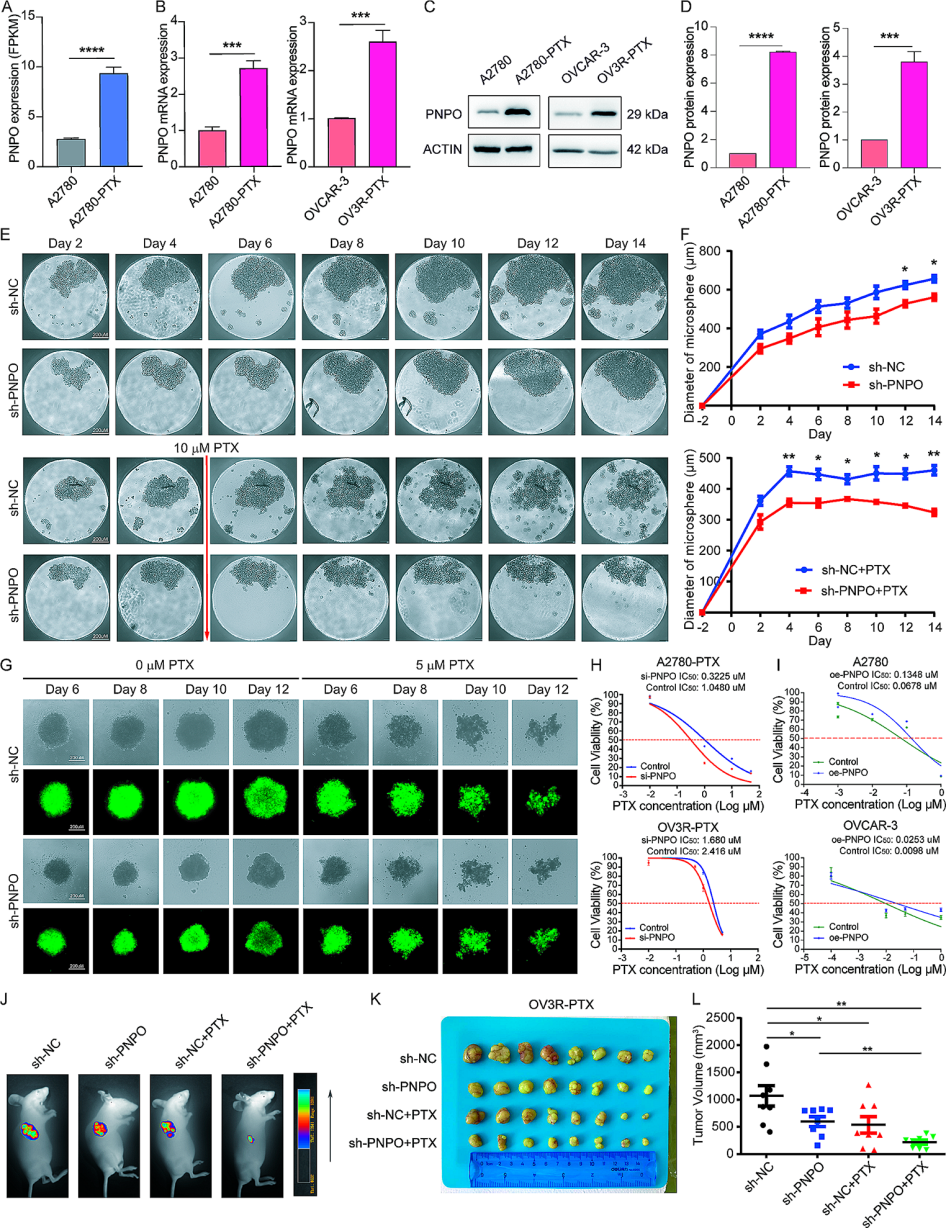
The enhancement of paclitaxel sensitivity by the knockdown of PNPO. **A** Expression of PNPO mRNA detected by RNA-sequencing. PNPO was upregulated in PTX-resistant OC cells (A2780-PTX) compared with its parental sensitive cells (A2780). **B** Detection of PNPO mRNA by qRT-PCR in two paired ovarian cancer cell lines (A2780 vs. A2780-PTX and OVCAR-3 vs. OV3R-PTX). Data were presented as mean ± SD (*n* = 3). **C** Detection of PNPO protein by Western blot in A2780, A2780-PTX, OVCAR-3, and OV3R-PTX cells. Representative images are shown. **D** Semi-quantitive analysis of protein bands from C. Data were presented as mean ± SD (*n* = 3) **E** Detection of microsphere in 3D culture by a microscope. OV3R-PTX cells were infected with negative control-shRNA (sh-NC) or PNPO-shRNA (sh-PNPO). The upper and lower panels presented the 3D culture of sh-NC and sh-PNPO cells in the absence or presence of 10 µM PTX. Scale bar, 200 μm. **F** Measurement of microsphere in 3D culture.The statistical analysis of the diameter of microspheres (*n* = 3). **G** Detection of spheroids of OV3R-PTX with or without sh-PNPO in the absence or presence of 5 µM PTX. Green showed GFP-positive PNPO-shRNA-infecting cells. Scale bar, 200 μm. **H** Measurement of the IC_50_ of PTX in si-NC or si-PNPO-transfected A2780-PTX and OV3R-PTX cells after PTX treatment. Data were presented as mean ± SD (*n* = 3). **I** Measurement of the IC_50_ of PTX in oe-NC or si-PNPO-transfected A2780 and OVCAR-3 cells after PTX treatment. Data were presented as mean ± SD (*n* = 3). **J** Detection of fluorescent tumor burden in the xenographic mouse model. Animals implanted with either sh-NC or sh-PNPO-infected OV3R-PTX cells were administrated with PTX at 0 or 15 mg/kg. Representative mice images were shown. **K** Measurement of tumor size in OV3R-PTX cell-transplanted mice. **L** Statistical analysis of tumor volume (*n* = 8). P values were calculated by the two-sided unpaired Student’s *t*-test (A, B, D, and F) and by the one-way ANOVA followed by Tukey’s multiple comparisons tests (L). Nonlinear regression was used to calculate the IC_50_ (H and I). IC_50_, the half-maximal inhibitory concentration; PTX, paclitaxel; ns, not significant; *, *P* < 0.05; **, *P* < 0.01; ***, *P* < 0.001; ****, *P* < 0.0001

By analyzing the chemoresistant data from the NCI-60 database, we found that the expression of PNPO was related to chemo-drug sensitivity. Cells with high expression of PNPO had a higher IC_50_ of cisplatin, docetaxel, and doxorubicin (Fig. S9A-C), indicating a potential role of PNPO on chemoresistance. RNA-seq showed that PNPO expression was higher in A2780-PTX than in A2780 cells (Fig. 8A). The overexpressed PNPO was confirmed in two PTX-resistant cells (A2780-PTX and OV3R-PTX) compared to their counterpart PTX-sensitive cells (A2780 and OVCAR-3) at mRNA levels (Fig. 8B) and protein levels (Fig. 8C-D) by qRT-PCR and Western blot, respectively. Next, we generated a PNPO-knocking down cell line by infecting OV3R-PTX cells with PNPO-shRNA (sh-PNPO). A negative control-shRNA cell line (sh-NC) was also constructed. The three-dimensional (3D) culture showed that PNPO-shRNA decreased the microsphere size of OV3R-PTX cells (Fig. 8E-F), indicating the suppression of cell growth by the knockdown of PNPO. Spheroid formation assays demonstrated that the knockdown of PNPO sensitized OV3R-PTX cells to PTX (Fig. 8G). Silencing PNPO decreased the IC_50_ of PTX in both PTX-resistant cells (A2780-PTX and OV3R-PTX) (Fig. 8H), whereas overexpressing PNPO increased the IC_50_ of PTX in both PTX-sensitive cells (A2780 and OVCAR-3) (Fig. 8I). These data suggest that the downregulation of PNPO may reverse the PTX resistance of OC cells. Next, we examined the influence of PNPO-shRNA on tumor formation in nude mice. The typical picture of an optical image presented a smaller tumor in sh-PNPO + PTX mice (Fig. 8J). Knockdown of PNPO suppressed tumor growth and tended to have the potential to augment in the presence of PTX (Fig. 8K) although no statistical difference between the sh-NC + PTX and sh-PNPO + PTX groups (Fig. 8L).

## Discussion

Human OC seriously threatens women’s lives and resistance to chemotherapy is a common cause of the progression of OC. The current study demonstrated for the first time that PNPO affects OC progression via autophagic flux and is a PTX-resistant factor. The knockdown of PNPO inhibited autophagic flux by influencing the function and distribution of lysosomes, leading to the suppression of tumor formation and the enhancement of PTX sensitivity in vitro and in vivo.

Our previous works have found the tumorigenic function of PNPO in various cancers [20]. Some mechanism studies revealed that the TGF-β signaling pathway negatively regulates the expression of PNPO at least in part via upregulating miR-143-3p in OC cells [7] and that the effects of PNPO on cell proliferation, migration, and invasion are mediated by MALAT1 through miR-216b-5p regulation in human breast invasive ductal carcinoma [8]. The present study further confirmed PNPO overexpression in OC and the positive correlation with OC cell proliferation via the regulation of cyclin B1 and phosphorylated CDK1, resulting in a shortening of the G2M phase in a cell cycle. Indeed, bioinformatic analysis also showed that high expression of PNPO was related to the cell cycle and lysosome enrichments.

It has been reported that the dysfunction of lysosomes and the unbalance of autophagy are associated with the progression of diseases, including cancers, infections, autoimmune, metabolic, neurodegeneration, cardiovascular disorders, kidney disease, etc. [21–26]. The present study proved that overexpressed PNPO enhanced the biogenesis and perinuclear distribution of lysosomes and thus increased the degradation of autophagosomes and enhanced the autophagic flux in OC cells. It was previously reported that the perinucleus distribution of lysosomes is favorable for the degradation of autophagosomes [27]. Emerging studies also reveal that defective autophagosome-lysosome fusion processes are associated with tumorigenesis and the robust lysosomes are critical for the degradation of autolysosomes, the late stage of autophagy [28, 29]. Generally, in the late stage of the process of autophagy, the lysosomes are infused with autophagosomes, contributing to the degradation of the content of autophagosomes [30]. Moreover, retrograde transport of lysosomes is recognized as a critical autophagy regulator and the perinuclear distribution is favorable for the fusion of autophagosome and lysosome [31, 32]. Current studies indirectly testify that PNPO influences autophagy during OC progression.

Interestingly, we showed that PNPO affected the late stage of autophagy but not the initiation of autophagy by monitoring the expression of LC3 and the colocalization of mCherry and GFP (mCherry-GFP-LC3) under starvation and CQ treatment. Our experiments demonstrated that PNPO promotes autophagy flux partially by enhancing the function of the lysosomes and CQ can block the effect of PNPO on autophagy by impairing the acidity of the lysosome and hence, increasing the degradation of autolysosomes. Our Western blot demonstrated that the silence of PNPO increased the expression of LC3-II. However, the increased LC3-II is not simply explained by the increase of autophagy because autophagy is a dynamic process and LC3-II can be degraded along with the degradation of lysosomes, which means both the formation of autophagosome and the impaired lysosomal degradation can lead to the increase of LC3. It has been reported that dormant tumors from autophagy-defective animals reactivated after being transplanted into autophagy-intact animals, indicating that autophagy supports tumor proliferation by recycling nutrients [33]. In the current study, we used HBSS as an autophagy inducer and CQ as an autophagy inhibitor to test the effect of PNPO on autophagic processes. CQ is known to inhibit the hydrolase of the lysosome by increasing the pH of lysosomes [34], which means lysosomes cannot perform a degradative function and therefore the degradation of autophagosome was blocked. The addition of CQ rescued the influence of PNPO on the expression of LC3-II, suggesting that PNPO regulates autophagy by influencing the degradation of autophagosomes.

Concerning the cellular protective function of autophagy in cancers, inhibitors by targeting autophagy are an emerging approach for cancer treatment [35]. Among these, CQ is regarded as a promising management for malignancy treatment and chemo-resistance. CQ has been included in various clinical trials [35] and the combination of CQ with other chemotherapy drugs has better performance than monotherapy including paclitaxel and gemcitabine in pancreatic cancer [36]. However, limitations still exist in the clinical application of using autophagy inhibitors. There are no accurate biomarkers that can reflect the autophagic levels with the tumors. In addition, it is hard to decide the dosage of CQ because of toxicity and side effects [37]. Thus, the development of an autophagy inhibitor with low toxicity is important. Here, the knockdown of PNPO and its combination with CQ significantly decreased the growth of tumors in vitro and in vivo, providing a potential application for the treatment of OC.

Currently, there is controversy about the contribution of autophagy to cancers [38]. As an intracellular balancing process in the face of acute stress, autophagy protects cells from unwarranted cell death. We found that LAMP2, an indicator of lysosomes [39], was upregulated in OC cells, and silencing LAMP2 suppressed OC cell growth and induced OC cell apoptosis. It has been shown that TFEB is a transcript factor of LAMP2 [40] and is responsible for the biogenesis of lysosomes [41]. Phosphorylated TFEB binds with YWHA/14-3-3 and is retained in the cytoplasm, while unphosphorylated TFEB translocated into the nucleus and binds to the CLEAR element on lysosome-related genes to promote the transcriptional expression-of these genes [42]. The current study demonstrated that PNPO did not regulate the expression of TFEB but promoted the translocation of TFEB, thereby inducing the transcription of lysosome-related genes such as LAMP2, leading to an increased biogenesis of lysosomes. Increased nuclear traslocation of TFEB rescues the autophagic flux damage and lysosome dysfunction [43]. Moreover, PNPO also upregulated the expression of CTSG, an indicator of the enzyme activity of lysosomes, representing the degradative ability of lysosomes [44].

Furthermore, we defined that PNPO is a PTX-resistant factor, which was overexpressed in PTX-resistant OC cells. The knockdown of PNPO sensitized the effect of PTX in vitro and in vivo. It is the first time to show that PNPO is involved in the chemoresistance in OC cells and the overexpression of PNPO may promote OC cell resistance to PTX. It has been reported that autophagy can increase PTX resistance in cancer [45]. As a key enzyme in the metabolism process of vitamin B6, PNPO was proven to promote the production of pyridoxal 5’-phosphate (PLP), an active form of vitamin B6. Our previous study showed that increased PLP suppressed the expression of PNPO, forming a negative feedback loop in OC cells [7]. Data from another laboratory revealed an inverse relationship between cancer risks and the intake of vitamin B6 and PLP in different types of cancers but may vary in supporting the protective role of vitamin B6 against cancer [46]. Therefore, targeting PNPO can be a potential therapeutic application for OC treatment and overcoming PTX resistance.

**Fig. 9:**
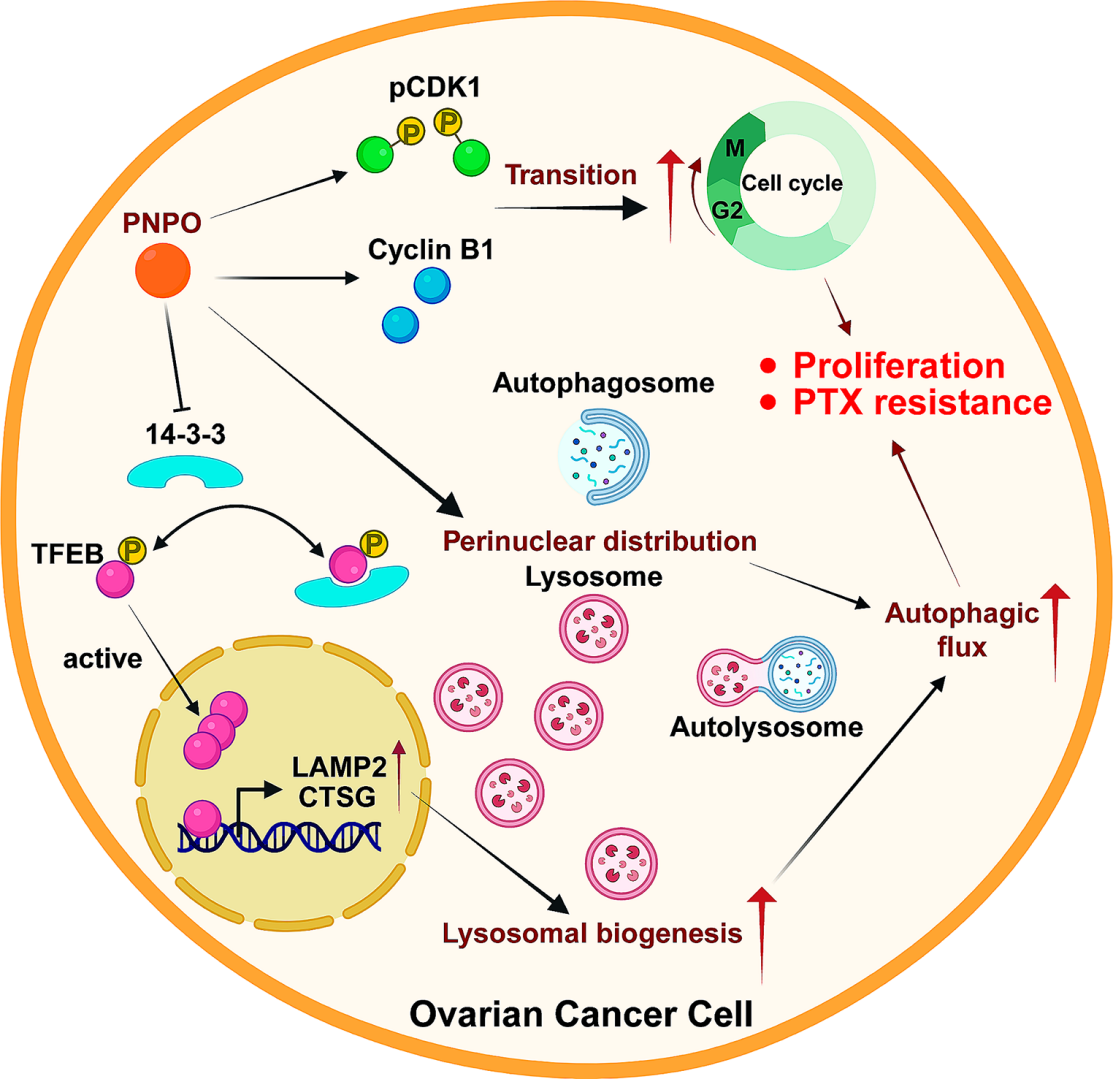
Schematic illustration of PNPO regulatory mechanism in ovarian cancer cells

In conclusion, the current study demonstrated the regulatory mechanism of PNPO on autophagic processes in OC progression (Fig. 9). PNPO promotes the survival of OC cells partially by regulating cell cycle-related protein cyclin B1 and phosphorylated CDK1, promoting the transition of the G2M phase and the growth of OC cells. PNPO induces the perinuclear distribution of lysosomes and nuclear translocation of TFEB by decreasing 14-3-3, which in turn enhances the biogenesis of lysosomes by increasing the expression of LAMP2, CTSG, etc. These alterations of lysosomes contribute to the enhancement of autophagic flux in OC cells. The knockdown of PNPO significantly suppresses the tumor formation in vitro and in vivo. In addition, PNPO is involved in the PTX resistance of OC cells, and the suppression of PNPO increases PTX sensitivity in vitro. Thus, PNPO has a regulatory effect on lysosomal biogenesis that in turn promotes autophagic flux, leading to OC cell proliferation, tumor formation, and PTX resistance. These data suggest that PNPO is a PTX-resistant factor and may imply a potential application by targeting PNPO as an autophagic inhibitor to suppress tumor growth and reverse PTX resistance in OC.

## Electronic supplementary material

Below is the link to the electronic supplementary material.

Supplementary Material 1

Supplementary Material 2

## Data Availability

No datasets were generated or analysed during the current study.
