# Correction: Targeting PNPO to suppress tumor growth via inhibiting autophagic flux and to reverse paclitaxel resistance in ovarian cancer

**DOI:** 10.1007/s10495-024-01998-7

**Published:** 2024-08-04

**Authors:** Xin Li, Wencai Guan, Huiqiang Liu, Jia Yuan, Fanchen Wang, Bin Guan, Junyu Chen, Qi Lu, Lingyun Zhang, Guoxiong Xu

**Affiliations:** 1 Research Center for Clinical Medicine, Jinshan Hospital, Fudan University, 1508 Longhang Road, Shanghai, 201508 China; 2 Department of Oncology, Shanghai Medical College, Fudan University, Shanghai, China; 3 Department of Obstetrics and Gynecology, Jinshan Hospital, Fudan University, Shanghai, China; 4 Department of Medical Oncology, Shanghai Geriatric Medical Center, Shanghai, China; 5 Department of Medical Oncology, Zhongshan Hospital, Fudan University, Shanghai, China; 6 Center for Tumor Diagnosis and Therapy, Jinshan Hospital, Fudan University, Shanghai, China

**Correction to: Apoptosis**

10.1007/s10495-024-01956-3.

In this article, the order that the authors appeared in the author list was incorrect. The correct order should be:

**Xin Li**^**1,2**^** · Wencai Guan**^**1**^** · Huiqiang Liu**^**1,2**^** · Jia Yuan**^**1,2**^** · Fanchen Wang**^**1,2**^** · Bin Guan**^**1,2**^** · Junyu Chen**^**1,2**^** · Qi Lu**^**1,2,3**^** · Lingyun Zhang**^**4,5**^** · Guoxiong Xu**^**1,2,6**^.

The original article has been corrected.

