# Supplementary material for: Targeting PNPO to suppress tumor growth via inhibiting autophagic flux and to reverse paclitaxel resistance in ovarian cancer
Source: Apoptosis. 2024 Apr 13;29(9-10):1546–63. doi: 10.1007/s10495-024-01956-3 (PMC11416418; doi:10.1007/s10495-024-01956-3)
Supplement: Supplementary file 1 — Supplementary Material 1 [file 10495_2024_1956_MOESM1_ESM.pdf]

Supplementary Tables:

Table S1. Sequences of siRNA

Items	Sequences	Position
siRNA		
PNPO-siRNA-1	GACTGGCTCTATGAGAGACtt	862-880
PNPO-siRNA-2	GGUGAUGGAGUUCUGGCAAtt	747-765
LAMP2-siRNA-1	CAGCAUGUAUUUGGUUAAUtt	1030-1048
LAMP2-siRNA-2	CUGGAGCAUUUCAGAUAAAtt	1146-1164
LAMP2-siRNA-3	GCAGCAUCUACUUAUUCAAtt	440-458
NC-siRNA	UUCUCCGAACGUCGUCAGUtt	Scramble

Gene ID of PNPO: 55163. Gene ID of LAMP2: 3910. NC, non-specific control.

Table S2. Sequences of PCR primer

Genes	Sequences
LAMP1-F	TCTCAGTGAACTACGACACCA
LAMP1-R	AGTGTATGTCCTCTTCCAAAAGC
PSAP-F	ATGCAAAGACGTTGTCACCG
PSAP-R	GGGAGGTAGGAGTCCACTATCT
CTSA-F	GTCGCCCAGAGCAATTTTGAG
CTSA-R	TCTCCCCGGTCAGGAAAAGTT
CTSG-F	GAGTCAGACGGAATCGAAACG
CTSG-R	CGGAGTGTATCTGTTCCCCTC
ATP6AP1-F	CAGCGACTTGCAGCTCTCTAC
ATP6AP1-R	TGAAATCCTCAATGCTCAGCTTG
ATP6V1E1-F	TTGCCACCAAAAACGATGTT
ATP6V1E1-R	AACTTCCTGTTGGCATTTGC
ACTIN_F	TCATCACCATTGGCAATGAG
ACTIN_R	CACTGTGTTGGCGTACAGGT
LAMP2-F	AAACCAGAAGCTGGAACCTATTCA
LAMP2-R	GAAGCAACCTTATCCTGAGTGATGT

F, forward primer; R, reverse primer.

Table S3. Information on the GEO datasets involved in the analysis of the Kaplan-Meier Plotter

GEO series	Contributors, Year	EOC (n)	GEO Platform #
GSE17260	Yoshihara K etc, 2010	110	GPL6480
GSE32062	Yoshihara K etc, 2012	270	GPL6480

Samples used for the prognostic analysis of PNPO were extracted from different GEO datasets. EOC, epithelial ovarian cancer; GEO, Gene Expression Omnibus; GPL, GEO Platform; n, number of samples.
