# Supplementary material for: Targeting PNPO to suppress tumor growth via inhibiting autophagic flux and to reverse paclitaxel resistance in ovarian cancer
Source: Apoptosis. 2024 Apr 13;29(9-10):1546–63. doi: 10.1007/s10495-024-01956-3 (PMC11416418; doi:10.1007/s10495-024-01956-3)
Supplement: Supplementary file 2 — Supplementary Material 2 [file 10495_2024_1956_MOESM2_ESM.pdf]

Supplementary Figures

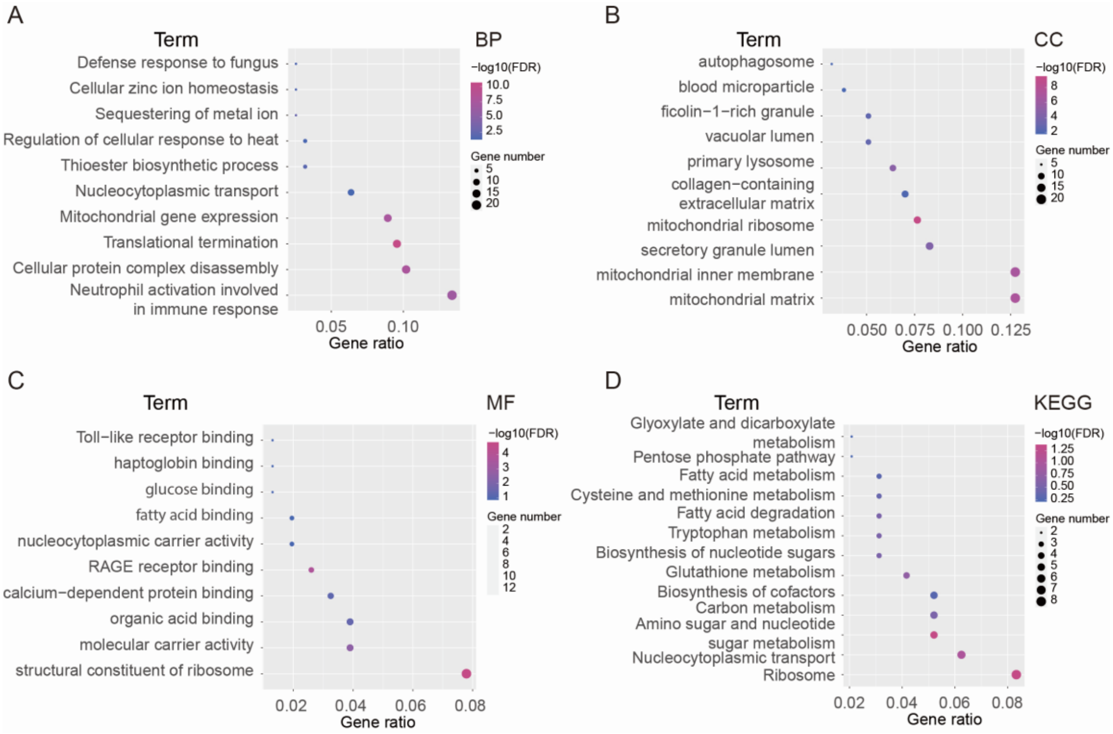

Figure S1 The biological function analyses of PNPO. (**A-C**) Gene Ontology enrichment analyses of PNPO. BP, biological process; CC, cellular component; MF, molecular function. (**D**) KEGG pathway enrichment analysis of PNPO.

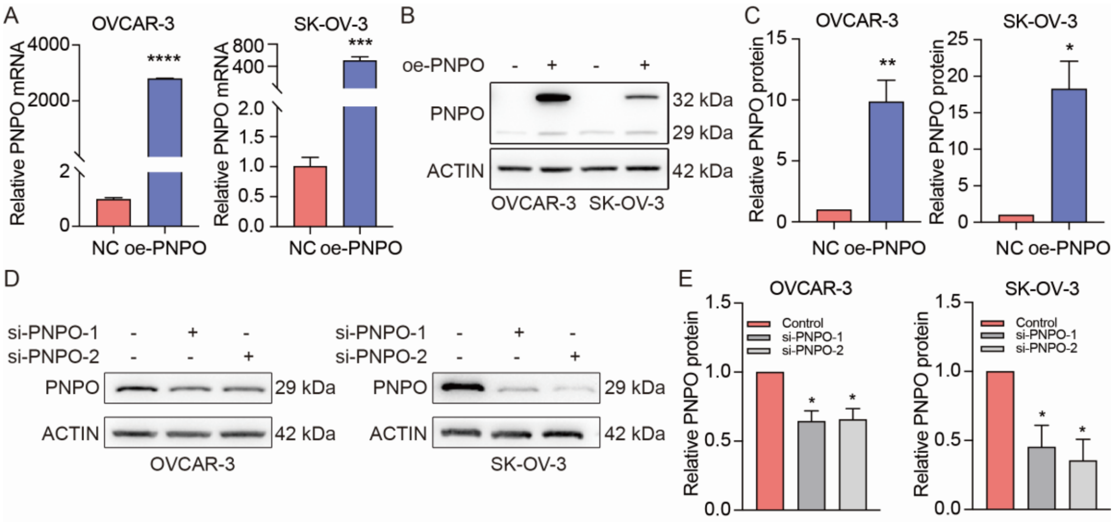

Figure S2 Expression of PNPO after si-PNPO and oe-PNPO transfection in OVCAR-3 and SK-OV-3 cells. (**A**) Detection of PNPO mRNA by qRT-PCR after transfecting PNPO-overexpressing plasmid. (**B**) Detection of PNPO protein by Western blot after transfecting PNPO-overexpressing plasmid. Representative images are shown. (**C**) Semi-quantitive analysis of protein bands from B. (**D**) Detection of PNPO protein by Western blot after transfecting 2 PNPO si-RNAs. Representative images are shown. (**E**) Semi-quantitive analysis of protein bands from D. Data were presented as mean ± SD (n=3). *, p<0.05; **, p<0.01; ***, p<0.001; ****, p<0.0001.

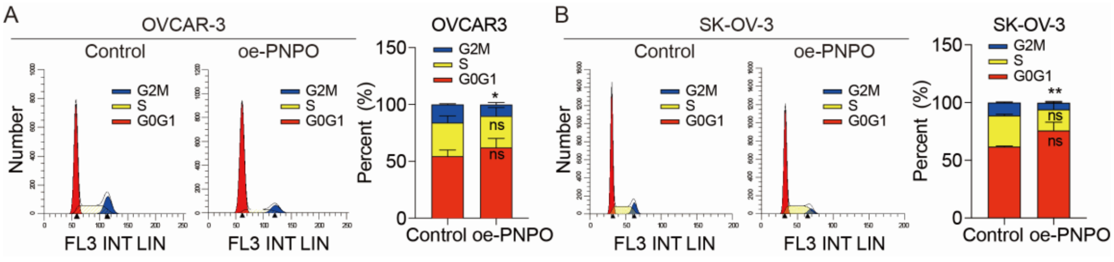

Figure S3 Effect of PNPO on the cell cycle. (**A-B**) OVCAR-3 and SK-OV-3 cells were transiently transfected with oe-PNPO plasmid for 48 h. The cell cycle was measured by the flow cytometry. Assays were repeated at least three times. Data were presented as mean ± SD. *, p<0.05; **, p<0.01.

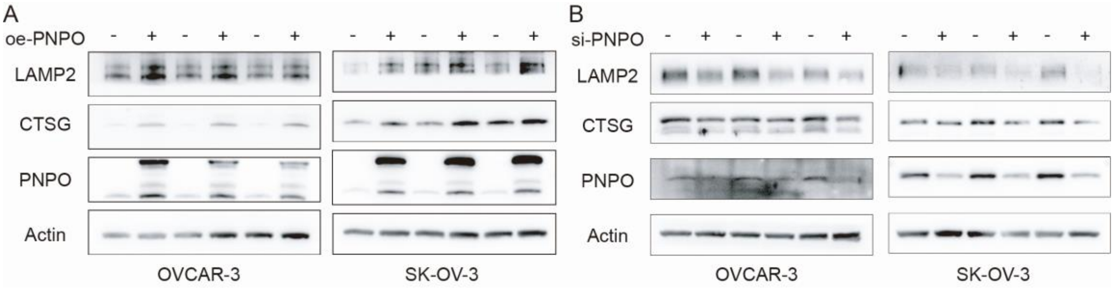

Figure S4 Effect of PNPO on the expression of lysosome-related proteins in ovarian cancer cells detected by Western blot. (**A**) Detection of LAMP2, CTSG, PNPO, and Actin proteins in cells transfected with PNPO-overexpressing plasmid. (**B**) Detection of LAMP2, CTSG, PNPO, and Actin proteins in cells transfected with PNPO si-RNA. Actin was used as a loading control.

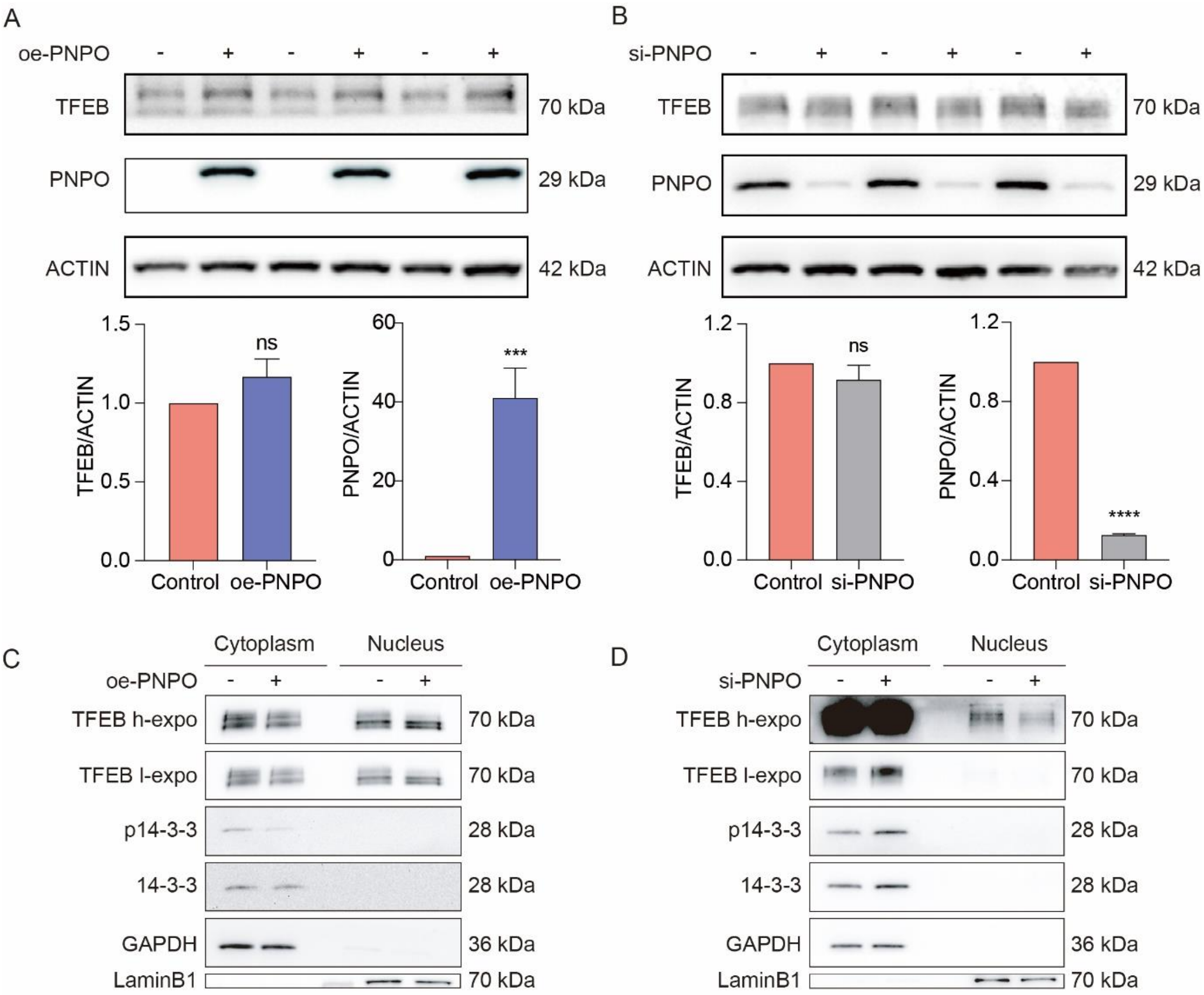

Figure S5 Regulatory effect of PNPO on the nuclear translocation of TFEB and 14-3-3 proteins SK-OV-3 cells. (**A-B**) Detection of total TFEB protein in cell lysates by Western blot. SK-OV-3 cells were transfected with PNPO-overexpressing plasmid or with PNPO si-RNA. The semi-quantitative analyses of protein expression were shown in the histograms. Data were presented as mean ± SD (n=3). ns, not significant; ***, $P < 0.001$; ****, $P < 0.0001$. (**C-D**) Detection of TFEB, phosphorylated 14-3-3 (p14-3-3), and pan 14-3-3 proteins in SK-OV-3 cells transfected with PNPO-overexpressing plasmid or with PNPO si-RNA. GAPDH and Lamin B1 were used as controls. TFEB h-expo, high exposure of TFEB membrane; TFEB l-expo, low exposure of TFEB membrane.

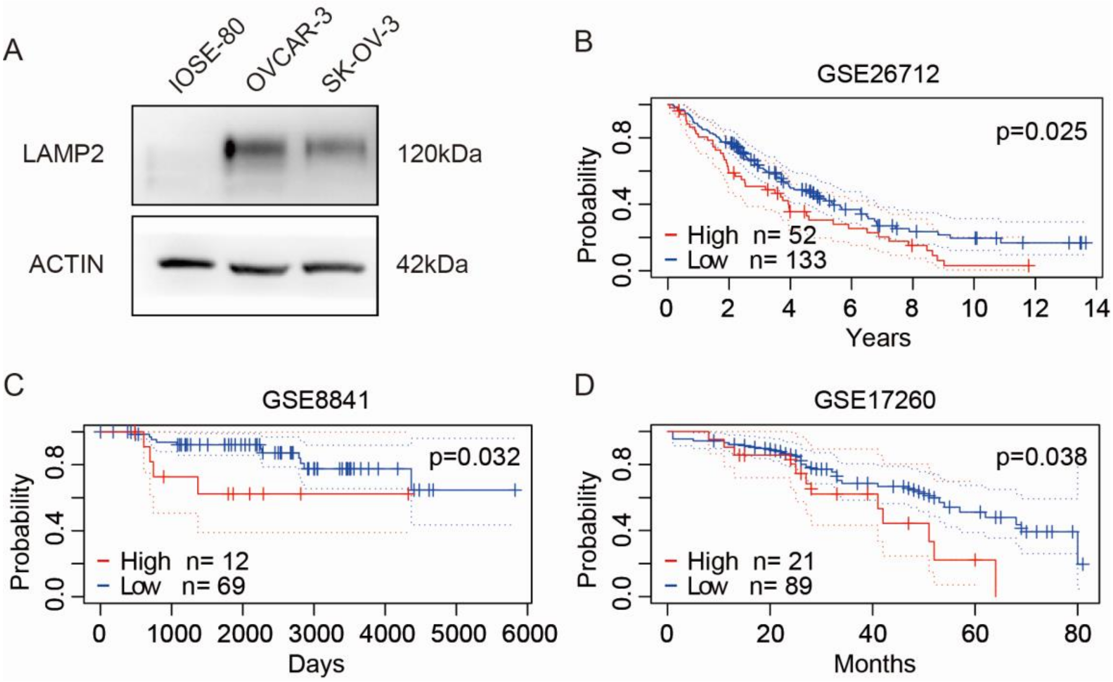

Figure S6 Expression of LAMP2 and prognostic analysis. (**A**) Detection of LAMP2 protein in IOSE-80, OVCAR-3, and SK-OV-3 cells by Western blot. (**B**) Kaplan-Meier plot analysis of GSE26712, GSE8841, and GSE 17260 datasets. High expression of LAMP2 was associated with poor prognosis of patients with ovarian cancer.

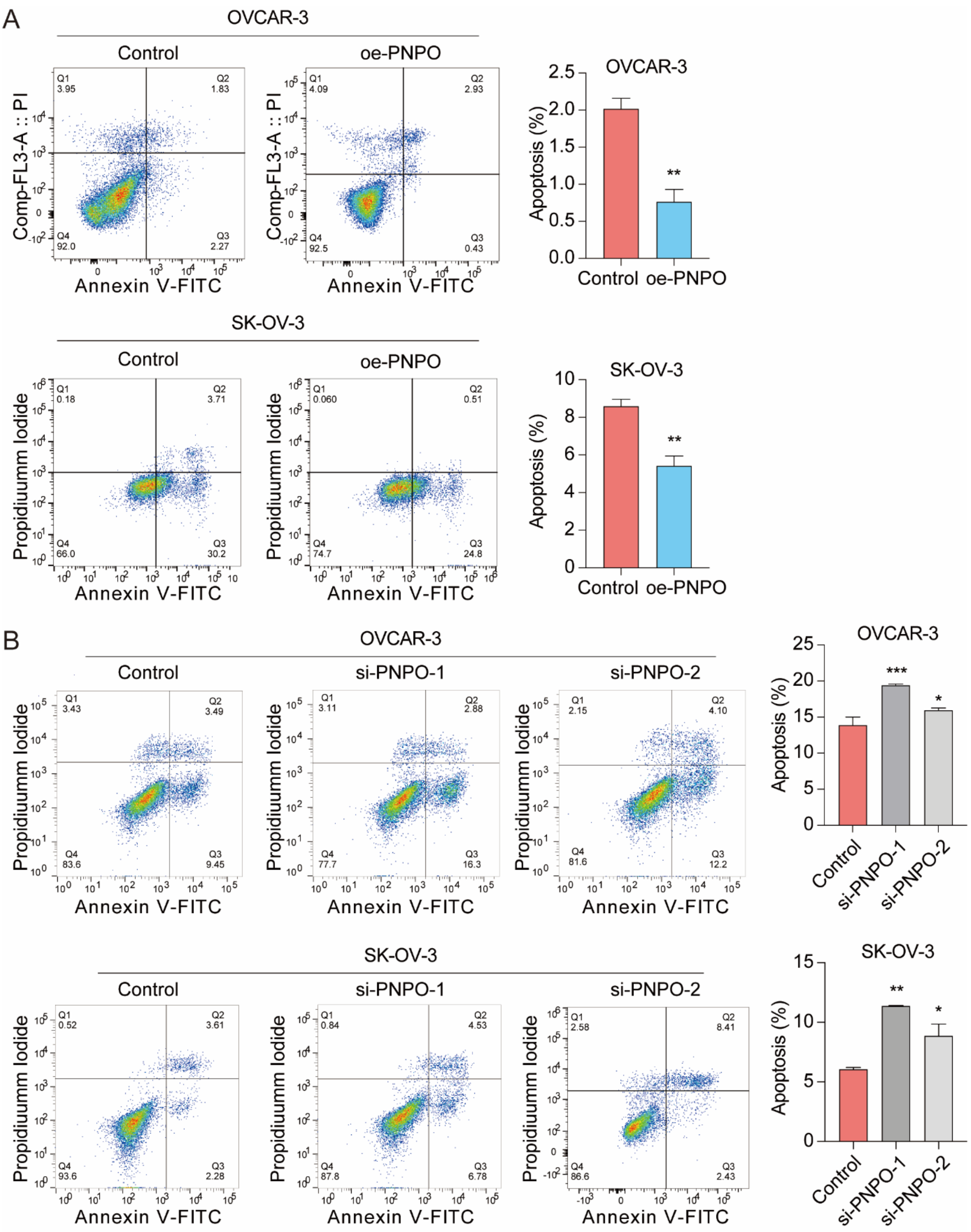

Figure S7 Detection of apoptosis by flow cytometry. (**A-B**) Control and oe-PNPO-transfected or si-PNPO-transfected OVCAR-3 and SK-OV-3 cells were seeded in a 6-well plate for 48 h in the presence or absence of si-LAMP2. Upregulation of PNPO significantly decreased apoptotic cells, whereas downregulation of PNPO increased the apoptotic cells. Data were presented as mean ± SD (n=3). *, P <0.05; **, P < 0.01; ***, P < 0.001.

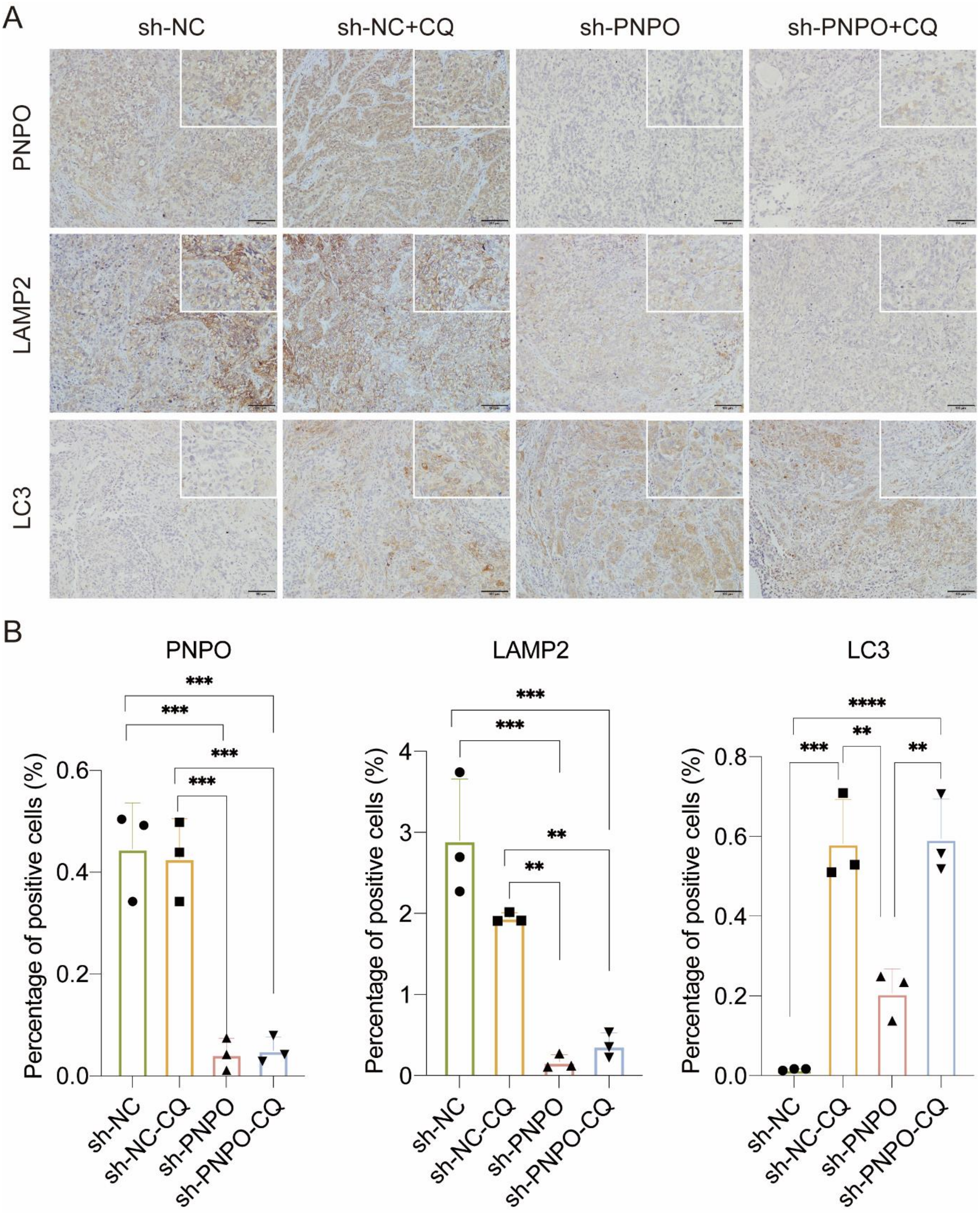

Figure S8 Analysis of the expression of PNPO, LAMP2, and LC3 protein in the tumor tissue. (**A**) Detection of the protein expression in the tumor tissue from the xenograft mouse model. by immunohistochemistry. Scale bar, 100 μM. (**B**) Statistical analysis of A by ImageJ IHC Profiler. The data were presented as percentages of positive cells. P values were calculated by the one-way ANOVA followed by Tukey's multiple comparisons test. **, $P < 0.01$; ***, $P < 0.001$; ****, $P < 0.0001$.

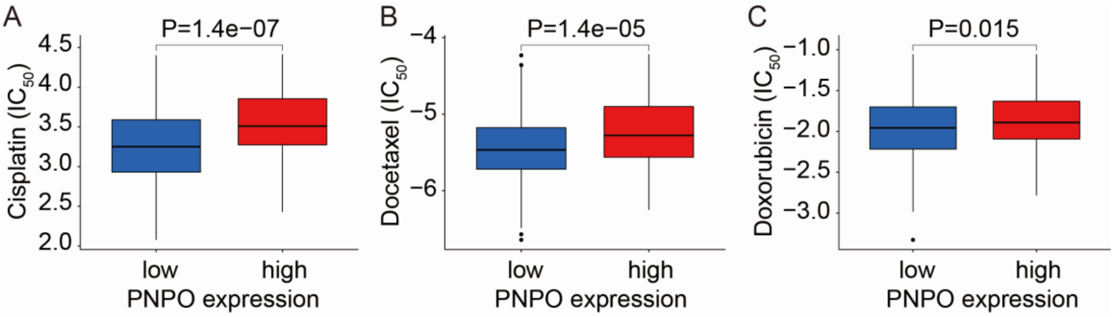

Figure S9 Detection of half-maximal inhibitory concentration (IC_{50}). (**A-C**) IC_{50} of cisplatin, docetaxel, and doxorubicin. High expression of PNPO increased the resistance in ovarian cancer cells.
